# Management Practices for the Control of *Haematobia irritans*, *Dermatobia hominis,* and *Cochliomyia hominivorax* in Cattle Across Latin America: A Sustainable, Collective Approach

**DOI:** 10.3390/pathogens15020177

**Published:** 2026-02-05

**Authors:** Roger I. Rodriguez-Vivas, Andre A. Cutolo, Antonio Thadeu M. de Barros, Ulises D. Cuore, Marcelo B. Molento, Sara López-Osorio, Daniel S. Rodrigues, Matias Spina, Fernando A. Borges, Welber D. Z. Lopes, Martín O. Pulido-Medellin, Cesar A. Fiel, Livio M. Costa-Junior, Oscar S. Anziani, Laura Marques San Martín, Gustavo A. Sabatini

**Affiliations:** 1 Campus de Ciencias Biológicas y Agropecuarias, Universidad Autónoma de Yucatán, Mérida 97000, Yucatan, Mexico; 2 Global Innovation, Boehringer Ingelheim Animal Health USA Inc., 6498 Jade Road, Fulton, MO 65251, USA; andre.cutolo@boehringer-ingelheim.com; 3 Embrapa Beef Cattle, Av. Rádio Maia 830, Campo Grande 79002-970, Brazil; thadeu.barros@embrapa.br; 4 Ex-Departamento de Parasitología, DILAVE, Montevideo 10000, Uruguay; ulises.cuore@gmail.com; 5 Laboratório de Parasitologia Clínica Veterinária, Departamento de Medicina Veterinária, Universidade Federal do Paraná—UFPR, Curitiba 81531-980, Brazil; molento@ufpr.br; 6 CIBAV Research Group, Department of Agricultural Sciences, School of Veterinary Medicine, Universidad de Antioquia U de A, Medellin 050010, Colombia; sara.lopezo@udea.edu.co; 7 Santa Rita Experimental Field, Empresa de Pesquisa Agropecuária de Minas Gerais, Prudente de Morais 35738-000, Brazil; dsrodrigues.epamig@gmail.com; 8 Boehrinher Ingelheim Animal Health Argentina, Cazadores de Coquimbo 2841 (Munro), Buenos Aires B1605EAA, Argentina; matias.spina@boehringer-ingelheim.com; 9 Laboratório de Doenças Parasitárias, Faculdade de Medicina Veterinária e Ciência Animal, Universidade Federal de Mato Grosso do Sul—UFMS, Campo Grande 79070-900, Brazil; fernando.borges@ufms.br; 10 Center for Veterinary Parasitology, School of Veterinary and Animal Science, Universidade Federal de Goiás, Goiania 74690-900, Brazil; wdzlopes@hotmail.com; 11 Veterinary Parasitology Laboratory, UPTC, Faculty of Agricultural Sciences, Pedagogical and Technological University of Colombia (UPTC), Tunja-Boyaca 150003, Colombia; mopm31@gmail.com; 12 Departamento de Sanidad Animal y Medicina Preventiva, Facultad de Ciencias Veterinarias, Campus Universitario, Universidad Nacional del Centro de la Provincia de Buenos Aires, Tandil 7000, Argentina; fiel.cesar.a@gmail.com; 13 Laboratory of Parasite Control, Universidade Federal do Maranhão—UFMA, Sao Luis 65080-040, Brazil; livio.martins@ufma.br; 14 Facultad de Ciencias Agropecuarias, Universidad Católica de Córdoba, Cordoba 5000, Argentina; anziani.oscar@gmail.com; 15 Ex Ministerio de Ganadería, Agricultura y Pesca (MGAP), Dirección General de Servicios Ganaderos (DGSG), Montevideo 11000, Uruguay; laura.marques.sm@gmail.com; 16 Global Strategic Marketing, Ruminant Antiparasitics, Boehringer Ingelheim Animal Health, 55216 Ingelheim am Rhein, Germany; gustavo.sabatini@boehringer-ingelheim.com

**Keywords:** *Haematobia irritans*, *Dermatobia hominis*, *Cochliomyia hominivorax*, livestock pest, control

## Abstract

Bovines are suitable hosts and can be affected by fly infestations. Flies pose a significant threat to cattle livestock in Latin America (LA), causing substantial economic repercussions to animal production (reduced productivity, veterinary expenses, and decreased animal welfare) and damage to human health. The most important flies affecting cattle in Argentina, Brazil, Colombia, Mexico, and Uruguay are *Haematobia irritans*, *Dermatobia hominis*, and *Cochliomyia hominivorax*. Due to production losses and the consequent economic costs associated with these flies, control measures must be implemented, primarily relying on insecticidal products. However, decision-making for preventing and treating animals with insecticides varies due to differences in environmental conditions across countries and regions, production systems, animal populations, infestation levels, animal welfare, and the prevalence of myiasis, among other factors. Although insecticides remain the most effective option for fly control in cattle, resistant populations have developed, rendering them less effective. To overcome fly resistance to insecticides, non-chemical (mechanical, environmental, biological, and genetic) methods are being integrated into alternative control and eradication strategies. The use of integrated livestock fly control contributes to safeguarding animal, public, and environmental health. This review is designed to support individuals and institutions, both civil and governmental, addressing the ongoing challenge posed by flies affecting livestock.

## 1. Introduction

Approximately 1.49 billion head of cattle are subject to ectoparasite infestation worldwide [1]. Arthropods, mainly dipterans (insects), mites, and ticks, are the most economically significant cattle ectoparasites. Approximately 150,000 species of dipterans have been described worldwide, with more than 8000 found only in the Americas. These insects play a crucial role in nature, as they are responsible for accelerating the decomposition of carcasses and other essential biological processes [2]. In general, the most critical flies affecting cattle health, production (weight gain and milk yield), and welfare in Latin America (LA) are *Haematobia irritans* Linnaeus, 1758), *Dermatobia hominis* (Linnaeus, 1781), and *Cochliomyia hominivorax* (Coquerel, 1858) and to a certain extent *Stomoxys calcitrans*. These parasites can have a significant economic impact on producers and local communities [1].

Except for *H. irritans*, all these dipterans infest warm-blooded animals, including humans. Primary damage can be caused by skin or tissue damage and blood loss. Several species of adult flies are hematophagous, causing intense irritation when they actively bite and, eventually, transmitting pathogens to cattle. Important damage also includes myiasis, as larvae invade and destroy the host’s living tissue to different degrees, and potentially leading to death as in the case of *C. hominivorax* larval infestation, allowing also for secondary infections. For example, during heavy infestations in cattle, *Haematobia irritans* can reduce growth rates, resulting in body weight losses of 300 g/day and 500 mL/day of produced milk, due to the “hassle factor” leading to reduced feed intake [3].

The biology and ecology (e.g., the dynamics of flight and attack) of flies that affect livestock are unique. Interactions between the farming system, epidemiological and climate aspects, and farm or herd hygiene can directly influence the incidence of ectoparasites and arthropod-borne vector diseases [1]. For this reason, veterinary practitioners must consider establishing good farming husbandry practices to prevent and control infestations in cattle [4]. Chemical insecticides continue to serve as the primary choice for the control of cattle-associated dipteran pests, forming the cornerstone of current management strategies in most production systems.

The most frequent classes of ectoparasiticides used to control flies in livestock are organophosphates (OPs), synthetic pyrethroids (SPs), phenylpyrazoles (PPs), insect growth regulators (IGRs), and macrocyclic lactones (MLs). Although their frequency and timing of use may vary depending on the geographical region, climatic area, and level of infestation, most drugs are used in preventive regimens with little or no technical criteria [5].

The use of acaricides and insecticides is beneficial for the control of arthropods but could cause harm to food safety, environment health, and resistance development. Chemical products can leave residues in animal products (e.g., meat, milk) that exceed official accepted limits when not managed properly, posing a risk to humans and the environment [5,6]. Contaminated food products may also impose trade and commercial restrictions, limiting market access for farmers worldwide. Moreover, the frequent use of these chemicals has accelerated the selection of resistance in fly populations. Therefore, methods combining other approaches for sustainable ectoparasite management need to be considered in the context of drug resistance, food quality and animal welfare and safety, herd selection for resilience and resistance, and environmental health [1].

Despite growing interest in alternative sustainable control strategies, chemical insecticides remain indispensable in managing cattle flies. Their rapid effect, broad-spectrum activity, and ease of application make them the most reliable option for achieving acceptable levels of fly control under field conditions. In many production systems, particularly those with high fly pressure or limited infrastructure for environmental interventions, chemical control is not only the most practical approach, but it is also often the only method capable of maintaining fly populations below economic thresholds. Without the use of insecticides, it is unlikely that producers could sustain adequate control, especially during peak infestation periods. Therefore, while integrated pest management and non-chemical methods are essential for long-term sustainability, chemical products continue to serve as the backbone of effective cattle fly control programs.

The present document outlines the best management practices for controlling flies that affect livestock in LA, supported by robust research and the authors’ field experience in Argentina, Brazil, Colombia, Mexico, and Uruguay. In cases where scientific information is lacking, reasonable collective recommendations are made based on the authors’ field-level experience and expertise.

## 2. A Brief Description of the Main Diptera Affecting Cattle in LA

*Haematobia irritans* (horn fly). This fly belongs to the family Muscidae and subfamily Stomoxyinae. The direct effects of *H. irritans* on cattle include blood loss, skin damage, and, most importantly, the constant restlessness of infested animals due to repeated bites, which cause a reduction in livestock performance. This fly is closely associated with cattle grazing in open pastures and rangeland. Larvae breed in undisturbed dung pats, and adult populations build up during the warmer months of the year [5,6], although in tropical regions, population peaks (infestation peaks) tend to depend on an interaction between temperature and rainfall, not necessarily occurring in the warmest months.

The irritation caused by the feeding process of the flies reduces grass consumption, interferes with rumination and food assimilation, and increases energy expenditure due to the intense and frequent defensive behaviors of infested animals. Horn flies exhibit a varying preference for cattle of different coat colors, breeds, genders, and ages, with a greater preference for adult dark-haired males. Thus, horn flies are more attracted to bulls than cows or steers, which increases with the age of the animals [7,8].

Although the number of flies on animals has been proposed as a criterion to support decisions about insecticide treatment in cattle herds, counting flies is a complex task to perform in practice by producers; in addition, the same number of horn flies can cause different effects on individual animals and is not necessarily indicative of stress [9]. The issue of establishing an economic threshold based on the infestation level is far from being a trivial challenge for horn flies. The marked variation in results across different regions and production systems makes it clear that there is no single index (economic threshold) suitable for all situations. Furthermore, performing individual fly counts on animals is unviable for producers and impractical in large herds of beef cattle. In practice, horn fly counts are not carried out on farms, and it seems utopian to most ordinary producers.

Fly infestation in a cattle herd is heterogeneous and depends on the individual susceptibility of each animal. In Brazil, Barros et al. [10] observed 50.3% of flies in 25% of the most infested animals in a Nelore herd, and Souza et al. [11] observed 66% of flies in 29.4% of animals in a mixed herd of European breeds. Such heterogeneous distribution has been attributed to characteristics associated with breed, coat color, skin temperature, hair density, secretions from sebaceous glands, and the success of fly feeding [8].

Generally, increases in infestation levels lead to altered behaviors (impacting animal welfare), at both a social and individual level. Such behaviors result from the state of tension and nervousness caused by flies in the animals, directly impacting cattle performance and welfare. The lateral movement of the head (head toss) is the most intense and characteristic defensive reaction in horn fly infestations. It may express a level of discomfort associated with a significant reduction in weight gain. In one study, the tactical treatment of cattle with a commercial insecticide product was recommended when approximately 25% of the herd exhibited a single head toss behavior within one minute of observation, which was associated with reduced weight gain [9].

**Figure 1:**
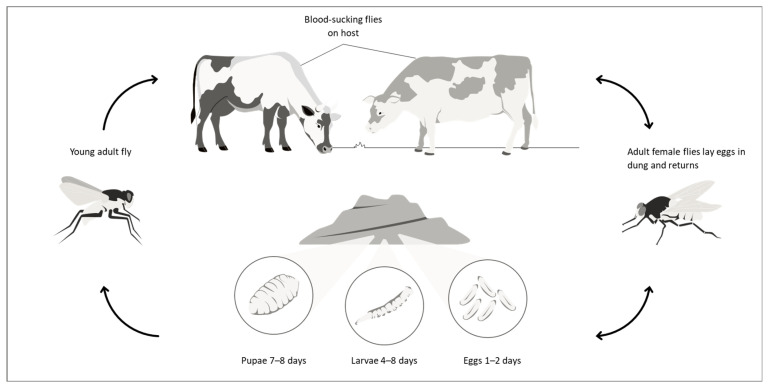
Life cycle of *Haematobia irritans*.

Another factor that influences the adverse effect of the horn fly on livestock is the quality and availability of pasture. Studies conducted in Brazil on the influence of horn flies on the weight gain of Nelore cattle from weaning to slaughter age showed that relatively low infestations (≤50 flies/animal) in situations of adequate pasture availability do not significantly affect weight gain and do not justify insecticide treatments. However, such infestations may dramatically reduce weight gain in conditions of food stress, such as drought [9]. Taking all this information together, we believe that herd behavior and pasture conditions should be considered when deciding on cattle treatment. However, it is important to emphasize that additional field studies are needed to provide the necessary reliability of this approach (head toss behavior check). The life cycle (Figure 1) and main biological characteristics of this fly are presented in Appendix A.

*Dermatobia hominis* (bot fly). This fly belongs to the family Oestridae and subfamily Cuterebrinae. The adult fly has atrophied mouthparts, and therefore it does not feed, receiving its nourishment only from food reserves accumulated during the larval stage. Females do not lay eggs directly on vertebrates but on smaller flies caught in flight and on whose abdomens the eggs are laid. Such transportation phenomena are known as phoresy, and egg-carrying insects are referred to as phoretic [12].

**Figure 2:**
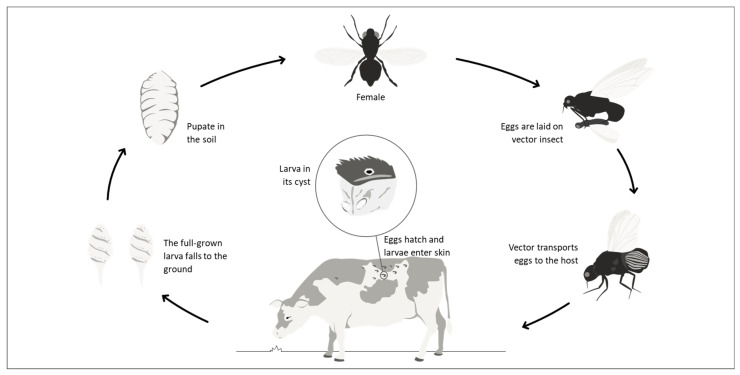
Life cycle of *Dermatobia hominis*.

After mating, the gravid female seeks a phoretic vector (e.g., *H. irritans*, *M. domestica*, *S. calcitrans*, etc.) to transport her eggs [13]. Once the phoretic is on a warm-blooded animal, larvae hatch and rapidly burrow through the skin and encyst to form a subcutaneous nodule, known as a warble. This furuncular myiasis (nodule formed under the skin) produces painful inflammation, fluid secretion, and lesions in the skin and subcutaneous tissue [13]. The life cycle (Figure 2) and main biological characteristics of this fly are presented in Appendix A.

*Cochliomyia hominivorax* (New World screwworm). This fly belongs to the family Calliphoridae and subfamily Chrysomyinae, and it is a parasite of mammals, including humans, during their larval stages. Larvae feeding on the skin and underlying tissues of the host cause a condition known as wound or traumatic myiasis, which can be fatal.

**Figure 3:**
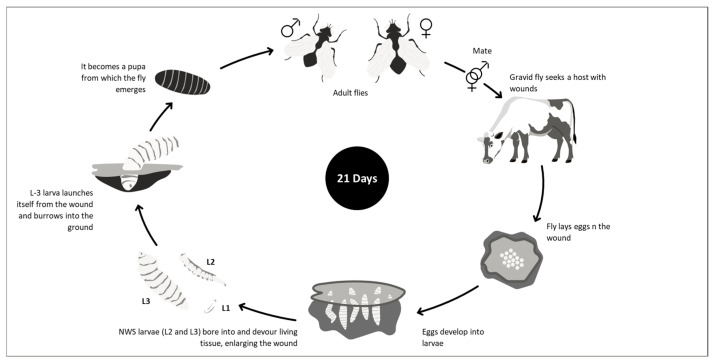
Life cycle of *Cochliomyia hominivorax*.

Unlike other calliphorids, *C. hominivorax* larvae feed on non-necrotic wounds, whereas necrophagous species like *C. macellaria* primarily consume necrotic tissues. *C. hominivorax* infestations are generally acquired at sites of previous wounding, due to natural causes or to animal husbandry practices. Still, they may also occur in the mucous membranes of the body orifices [14]. The life cycle (Figure 3) and main biological characteristics of this fly are presented in Appendix A [15,16,17,18,19,20,21,22,23,24,25,26,27,28].

## 3. Economic Losses of Flies on Cattle in LA

Flies can pose a significant problem for cattle, as evidenced by the following potential economic estimates:

*H. irritans.* This fly species feeds 20 to 38 times a day, consuming micro portions of blood in each feeding, with an average of 10 μL per day per fly. Chronic infestations of *H. irritans* result in dermal lesions and scarring on cattle hides, which compromise the integrity and aesthetic quality of the leather, thereby reducing its commercial value for the tanning industry. This fly species is the intermediate host of parasites (e.g., *Stephanofilaria stilesi*). However, the primary damage it causes is associated with its feeding process, which results in severe cattle irritation, loss of energy, altered grazing behavior, decreased weight gain, low conversion efficiency, and skin damage, among other effects [29].

In Mexico, this fly species affects cattle during 7 months of the year (from June to December). Based on Mexico’s potential at-risk cattle population (steers/heifers, cows, calves, and dairy cows), the estimated annual losses attributed to *H. irritans* infestation amount to USD 231.66 million [29,30]. In Brazil, horn fly infestations occur practically throughout the whole year, although they are much fewer during the dry season (winter). The economic impact attributed to horn flies in Brazil was estimated at USD 2558.32 million per year, based on an at-risk population of almost 190 million cattle [28]. At the national level, the economic impact of *H. irritans* has not been estimated in Argentina, Colombia, or Uruguay.

*C. hominivorax*. Myiasis caused by *C. hominivorax* is a significant cause of mortality among newborn calves [31], in addition to directly impacting milk production, fertility, feed conversion rates, and animal weight gain. The prevalence of myiasis in adult cattle is relatively lower than in newborns. In either young or adult animals, infestations are usually fatal if left untreated, with death occurring within 7 to 10 days after infestation [31].

The most significant economic impact of *C. hominivorax* in LA cattle production is associated with the prophylaxis and control of myiasis in natural or handling-caused wounds, actions that require an average of 20 h of work per month in each livestock establishment [32]. The losses caused by *C. hominivorax* in Uruguay are attributed to the work required to heal wounds at the field level (60%), veterinary supplies (8%), and mortality in sheep (20%) and cattle (12%) [33].

In Brazil, economic losses in cattle farming due to *C. hominivorax* are estimated at approximately USD 340 million [28], primarily resulting from calf mortality. However, this figure does not account for indirect costs, such as labor and treatment expenses. In dairy production systems with zebu and crossbred animals, calf mortality generates additional losses by compromising cow milk production. In Uruguay’s livestock farms, annual losses due to *C. hominivorax* myiasis were estimated at USD 43.1 million [34]. In Argentina, economic losses exceeded USD 50 million annually. However, this estimate does not include the decrease in the weight gain of developing cattle affected by *C. hominivorax* or the mortality of animals [32].

*D. hominis*. The myiasis caused by *D. hominis* larvae damages the skin and subcutaneous tissue of cattle, resulting in weight loss and reduced leather quality. Additionally, it affects prime areas that make up the industrially usable part of the animal’s hide [13]. Another potential, but still neglected, economic loss due to *D. hominis* is the impact in humans during tourism activities, especially ecotourism in LA. In this context, Brazil is the tourist destination with the highest number of reports of tourists infested with *D. hominis* larvae [35].

Brazil and Colombia have estimated the economic losses caused by *D. hominis* in cattle. In Brazil, the latest estimate of the economic losses caused by the parasite amounts to USD 383 million annually [20], based on the cattle population exposed to the parasite and the annual weight loss of 40.6 g caused by each larva, as well as the losses resulting from leather depreciation. The economic losses caused by *D. hominis* in Colombia were estimated at approximately USD 31 million per year [36].

## 4. Pathogen Transmission by Flies in Cattle

Although horn flies can mechanically transmit pathogens to cattle, they are not considered an epidemiologically important vector for most agents of vector-borne diseases affecting cattle, such as *Anaplasma marginale*, *Trypanosoma vivax*, and *T. evansi* [37]. Their vectorial importance has been attributed to the detection of pathogens in the proboscis or digestive tract; however, such findings only refer to a previous meal on an infected host, not necessarily leading to the effective mechanical transmission of the pathogen nor an essential role in the epidemiological chain of the disease. On the other hand, a relevant role of *H. irritans* has been evidenced in the epidemiology of mastitis in dairy cattle through the mechanical transmission (by contact) of *Staphylococcus aureus*. Additionally, *H. irritans* serves as the intermediate host of the nematode *Stephanofilaria stilesi* and acts as a phoretic vector for *D. hominis* eggs [38].

Neither *D. hominis* nor *C. hominivorax* is important in the transmission of pathogens or in the epidemiology of diseases affecting livestock; their actual importance lies in causing myiasis.

## 5. The Best Management Practices for the Sustainable Control of Flies That Affect Livestock in Argentina

### 5.1. An Overview of Cattle Demographics in Argentina

The primary production of bovine chains is represented by 130,803 agricultural farms that operate their herds in almost 400,000 premises involved in feedlot, breeding, rearing, and fattening, as well as about 13,000 bovine dairy farms. Currently, Argentina has a livestock herd, mostly raised on pasture, of almost 51.6 million head as of December 2024, of which some 3.16 million correspond to dairy cattle (1.56 million dairy cows), with a strong predominance of the Holstein breed (92%). The characterization of stocks of free-range fattening cattle reaches a total of 1.94 million cattle in 1126 establishments. Argentina produces about 3.13 million tons of meat (13.9 million heads), 70% of which is consumed by the domestic market (https://surdelsur.com/en/livestock-argentina/ accessed on 1 January 2026, National Agricultural Census 2002).

The Argentinian subtropical region is located north of 29° south latitude, separated from the temperate region by an ecotone that does not exceed 200 km in the southern direction. This limit approximately coincides with the isoline of the frost-free period longer than 280 days and where the last spring frosts arrive at the end of August or before. The predominant beef breeds—based on productive characteristics and adaptability to climate and parasitic diseases (e.g., the cattle tick)—are those of *Bos indicus* and their crosses with *Bos taurus*, with a clear predominance of the synthetic Bradford and Brangus breeds.

Concerning the cold-temperate region, Aberdeen angus is the most abundant breed due to its rusticity, meat production, and climatic adaptability. On the other hand, Hereford and Polled Hereford are widely spread throughout the country’s livestock zone, but their greatest relative participation is in the Patagonian region, mainly in the Patagonian foothills and on the southern island of Tierra del Fuego.

### 5.2. Epidemiology of Haematobia irritans

Geographic distribution. Since its introduction in 1991 in Argentina, *Haematobia irritans* quickly colonized the bovine production area of the northeast, northwest, and central part of the country and currently reaches northern Patagonia. In the north-central area of Argentina, where most of the national bovine herd is based, the horn fly begins to build important populations around October–November and decreases drastically with the first frosts [39].

**Figure 4:**
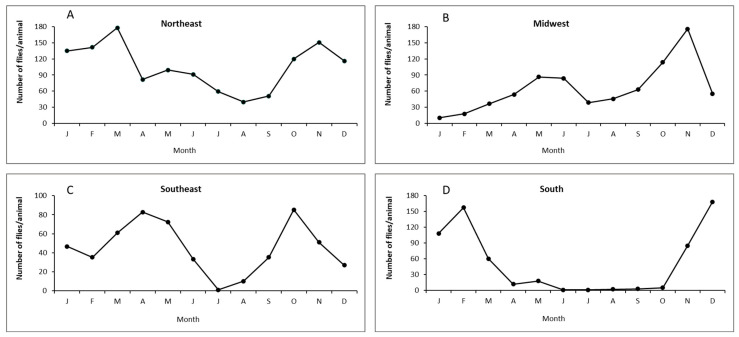
Population dynamics of *Haematobia irritans* in four regions of Brazil. (**A**) Northeast (Patos, PB), (**B**) Midwest (Campo Grande, MS), (**C**) Southeast (Araçatuba, SP), and (**D**) South (Eldorado do Sul, RS) [9,66,67,68].

Population dynamics. Population studies carried out for eight years (from November 1992 to August 2000) in the central area of Argentina (31°12′ S–61°29′ W) showed the presence of two population peaks in Holstein cows without treatments against ectoparasites (Figure 4), the first of these peaks towards the end of spring and the second one towards the beginning of autumn [40]. However, adults of *H. irritans* were consistently found during winter with greater numbers in late July–August. This 8-year study indicated that an unknown proportion of insects in immature stages were insensitive to factors that induce diapause and remained in the central area of Argentina during winter.

### 5.3. Epidemiology of Dermatobia hominis

Geographic distribution. In Argentina, *D. hominis* is distributed in the subtropical areas of the northeastern provinces, mainly Misiones, Corrientes, and Formosa, covering approximately 60,000 square km. *D. hominis* is also sporadically observed in the neighbor provinces of Santa Fe and Entre Ríos, located south of those mentioned above [41].

Population dynamics. In all cases, the presence of *D. hominis* is seasonal in spring and summer. The main host is cattle, and nodules with *D. hominis* larvae are preferably located in the anterior region of these animals, mainly on the back, scapular area, and ribs. The infestion of this fly is also an important zoonosis in Argentina where frequent cases in humans are observed in people living, working, or just visiting endemic areas [42]. In the province of Misiones, hides sent to the industry may be affected by parasitic nodules on 65% of their surface, but there are no regional or national estimates of the economic losses caused by *D. hominis* in Argentina.

### 5.4. Epidemiology of Cochliomyia hominivorax

Geographic distribution. In the area of Argentina located north of parallel 29° S, myiasis caused by *C. hominivorax* constitutes a health problem throughout the year. In these subtropical areas, the population growth rate of this insect is more related to the density of hosts, the availability of wounds for oviposition, and their healing rate than to climatic parameters, such as temperature. The tendency to produce a constant number of cases in subtropical regions was shown in a study carried out over three years in the northeastern region of Chaco [43], in which the prevalence of myiasis was very similar during the summer and winter months (23% and 21%, respectively). On the contrary, in the central area of Argentina, epizootics of myiasis caused by *C. hominivorax* showed a markedly seasonal pattern with a higher incidence of cases in the warmer months of the year and their absence during the winter [44].

Population dynamics. In national cattle production, *C. hominivorax* is considered the most economically important insect, and more than 95% of the cattle stock is currently found in areas where this insect is enzootic. In the humid and sub-humid pampas and in the semiarid pampa region, where 70% of the national cattle herd is found (approximately 36 million animals), the presence of *C. hominivorax* is limited to spring–autumn [45]. Meanwhile, in the subtropical areas of the northeast and northwest of the country (approximately 25% of the total cattle population), myiasis caused by this insect is a health problem throughout the year [43]. Temperature is the causal variable of these modifications in the populations of *C. hominivorax* (critical limit: 10 to 12 °C), and the low winter temperatures that occur below the 28° or 29° south latitude would explain the severe population changes. Thus, because this insect does not develop diapause phenomena, the cases of myiasis caused by *C. hominivorax* that occur in the form of seasonal enzootics in the central area of Argentina are mostly the result of expansive migrations from the subtropical regions of the country where this insect remains throughout the year [45]. These migratory phenomena constitute an important component in the survival strategy of this fly species, and its movements vary between 80 and 160 km within a generation. Starting in spring, the invasion of new territories in each successive generation would explain the rapid increase in the areas occupied by *C. hominivorax* in the central area of Argentina [45].

The climatic suitability of central Argentina to support the presence of permanent populations of *C. hominivorax* could be enhanced by an increase in annual temperatures. For example, predictive models indicate that an increase of about 2 °C in temperature could significantly extend the area of endemic dispersion of this insect in North America with movements from the south to the north [36,46].

### 5.5. Chemical Control of Haematobia irritans, Dermatobia hominis, and Cochliomyia hominivorax in Argentina

#### 5.5.1. Chemical Control of *H. irritans* in Argentina

Products available for their control. Since the introduction of this insect in Argentina (1991), until the first half of the 2000s, the pour-on application of SPs (mainly cypermethrin) was widely used in the country. Originally, these applications reported more than 80% efficacy for a period of approximately 60 days. However, the severe and widespread development of resistance to this chemical group throughout the country has led to the replacement of SPs with other insecticides such as OPs or, to a lesser extent, neonicotinoids [47]. Chemical groups available in Argentina to control cattle flies are indicated in Table 1.

(a) Adulticides. When treatments are specifically aimed at controlling *H. irritans*, OP is currently the most widely used chemical group, either in the form of insecticidal ear tags (ethion, chlorpyriphos, or diazinon) or in pour-on applications. The latter are generally presented as mixtures that include cypermethrin and piperonyl butoxide (PBO) as a synergist. In Argentina, there are also pour-on formulations containing imidacloprid plus cypermethrin. In general, OP ear tags present adequate persistence for a period of 14 to 16 weeks, while pour-on applications last 18 to 25 days [47]. In the case of the above-mentioned mixtures containing imidacloprid, such a period lasts for 30 to 35 days approximately [45].

Specifically, in dairy cows, the application of OP or carbamate pesticides through self-treatment bags has proven to be a practical, economical, and effective alternative control, but this form of treatment has been poorly adopted in Argentina. Organophosphate insecticides formulated in powder are generally safe and less toxic than liquid formulations such as emulsions [48].

(b) Larvicides. The different uses of larvicidal formulations neurotoxic to the insects, such as OP or IGR juvenile hormone analogs (methoprene) or those that inhibit or interfere with chitin synthesis (diflubenzuron), have been tested and are commercially available in the Argentinean veterinary market [47]. These products can be administered as feed additives, in water, or in intraruminal boluses (diflubenzuron). Their efficacy is very high but exclusively restricted to the immature stages that develop in the feces of treated animals.

(c) MLs and their adulticidal and larvicidal activity. The efficacy of long-acting injectable formulations of ivermectin (IVM) has also shown a reduction by more than 80% in fly numbers during the two weeks post-treatment when compared to untreated animals, and similar efficacies for 14 days have been reported with the use of pour-on eprinomectin on adult flies [49].

In the recent past, it has been recommended in Argentina to delay the administration of insecticides until observing averages of approximately 100 to 200 flies in lactating or beef cattle or when animals show signs of marked irritability independently of the parasite load. Currently, and specifically in grazing dairy cattle, it could be advisable to lower this threshold to less than 50 flies per animal to reduce the potential role of this insect as a vector of *Staphylococcus aureus* [45].

#### 5.5.2. Chemical Control of *C. hominivorax* in Argentina

In Argentina, the control of this dipteran is currently carried out exclusively on the larval stages by applying insecticides to the hosts [50]. Although the prevention and treatment of the New World screwworm myiasis are some of the most common practices in Argentinean cattle production, there is notoriously little documentation on the susceptibility of *C. hominivorax* to the different insecticide groups and formulations. One exception is the recent record of the resistance of *C. hominivorax* to doramectin (DRM) [51].

The residual action period of insecticides is a very important economic and management factor in the prophylaxis of susceptible wounds. In this context, there is an increasing use of avermectins for the prevention of navel myiasis in newborn calves or by common management practices such as castration, dehorning, etc. Macrocyclic lactones (MLs), including ivermectin, abamectin, and doramectin, exhibit high preventive and therapeutic efficacy against *C. hominivorax* larvae under conditions of susceptibility. Comparative studies indicate that during the initial phase of resistance development, doramectin may retain slightly higher efficacy than ivermectin. However, once resistance to macrocyclic lactones becomes established, cross-resistance among all members of the class is common, resulting in a marked reduction in or complete loss of efficacy against screwworm larvae [52]. Due to its activity against first-stage larvae, IVM and DRM protect susceptible wounds (for example, umbilical or castration wounds) for approximately 10 days. However macrocyclic lactones are hardly effective in wounds with the presence of second- and third-stage larvae. This low efficacy against active myiasis is not usually considered in many situations, and in Argentina, producers and veterinarians use MLs without differentiating between prophylaxis or treatments [32].

In cases of active myiasis, curative treatments are based on the topical application of pastes, liquids, powders, or sprays of mainly chlorpyriphos, coumaphos, dichlorvos, and fenitrothion, alone or in mixture with SPs, mainly cypermethrin. When applied topically, these insecticides cause the expulsion of larvae that generally die in the soil, although some of them may remain inside the wounds. The effectiveness of these forms of topical application is very variable and depends on the location and unevenness of the wounds and on factors that can facilitate the removal of insecticides such as bleeding, rain, and animal licking. Topical treatments also have a certain prophylactic action on susceptible wounds, but in general, their residual properties do not exceed two or three days [32].

Under extensive cattle production conditions, the currently suggested treatment for wounds with second- and third-stage larvae consists of the following: (a) the application of a topical insecticide to cause the immediate expulsion of the larvae and (b) the injectable administration of 0.2 mg/kg of DRM or pour-on application of fluralaner (introduced to the Argentine veterinary market in 2024) to protect the wound for 10 to 12 days and allow it to heal [53]. In dairy production, systemic insecticides such as avermectins or fluralaner, commonly used in beef herds due to their obvious practicality and long-lasting residual characteristics, are incompatible in lactating dairy cows or even in females with advanced pregnancy. The use of these drugs in dairy production is thus strictly restricted to the prophylaxis of susceptible wounds in calves or in dry pregnant cows before reaching the last third of gestation.

## 6. The Best Management Practices for the Sustainable Control of Flies That Affect Livestock in Brazil

### 6.1. An Overview of Cattle Demographics in Brazil

Brazil has the world’s largest cattle herd. The most recent reported data indicated that there are 238.6 million heads in more than 2.5 million farms [54]. Of this total, 80% of the animals are destined exclusively for beef cattle. Brazil is also the leading global beef exporter, accounting for 21% of traded beef [55]. Although it has a smaller herd, dairy farming also stands out internationally as it is the fifth largest milk producer, with a total of 15.74 million milked cows and a production of 36.74 billion liters per year [54].

Brazil’s bovine production systems are predominantly pasture-based. The pasture area covers 160 million hectares, with a stocking rate of 0.8 animal unit/ha [56].

The Midwest region accounts for the largest share (32%) of the Brazilian herd, with 62 million heads, followed by the north region, with over 50 million, and 60% growth in the last 20 years. Large herds are also observed in the south and southeast regions, where, in addition to significant beef cattle production, most of the dairy production takes place [57].

The southern region of the country is recognized as an area of lower temperatures and fertile soil types, which allows European cattle breeds to adapt well. Most of the animals used for meat production are Aberdeen Angus and Hereford, while those used for milk production are Holstein and Jersey [58].

For meat production in the rest of the country, zebu cattle predominate, with a very strong preference for Nelore cattle. So-called industrial crossbreeding, based on Nelore and Brahman cattle, has been gaining ground in technologically advanced systems [58]. For milk production, the Holstein, Gir, and Girolando breeds predominate. The latter is defined by the 5/8 Holstein x Gir breed proportion but still allows for wide variations in bloodline composition. Generally, the higher the level of intensification of the milk production system, the higher the proportion of Holstein blood [59].

### 6.2. Epidemiology of Haematobia irritans

Geographic distribution. The first record of *H. irritans* in Brazil dates to the early 1980s, specifically in the north of the country. However, its presence in the region dates to the mid-1970s [60]. Once making it across the Amazon River, the horn fly quickly dispersed throughout the country, facilitated by cattle transport routes, and reached southern regions by 1991, crossing international borders. Since then, the horn fly has adapted to different tropical and subtropical biomes and has become widely distributed throughout Brazil [61].

Population dynamics. In Brazil, horn fly infestations occur practically throughout the year, although they are much fewer during the dry season (winter) (Figure 4). Relatively fewer infestations appear to occur in dairy cattle, probably due to the management of animal waste and more frequent antiparasitic treatments against ticks and helminths. However, horn fly infestations cause significant problems in dairy cattle, and their impact still requires adequate assessment. The horn fly exhibits bimodal seasonality, with yearly population peaks occurring shortly after the beginning and near the end of the rainy season. Generally, the peak of the rainy season occurs between November and December, while the peak at the end of the rainy season typically falls between March and April. However, both peaks can be anticipated or postponed by annual macroclimatic trends, and it is not uncommon for their occurrence to be from October to December and from February to May, respectively. Thus, the long season of higher horn fly infestations in the country corresponds basically to the period from spring to autumn. On the other hand, the duration of horn fly peaks is relatively short, lasting two weeks or less [62]. *H. irritans* has a short biological cycle, with several generations per year in Brazil (e.g., 30 in Paraíba state, 22 in Mato Grosso do Sul state, and 19 in São Paulo state) [63,64,65]. The egg-to-adult development of horn flies in Brazil can vary from 7 to 11 days in the Northeast region and from 9 to 17 days in the Midwest [64,65].

### 6.3. Epidemiology of Dermatobia hominis

*Geographic distribution. D. hominis* is normally a parasite of wildlife animals in the Neotropical region and the larval parasitism in livestock animals was an adaptation to this new host, by the time domestic bovine species were introduced into the American continent. The latest scientific information on the geographical distribution of bot flies in Brazil was published more than 40 years ago, and through a comprehensive epidemiological survey conducted at the time, *D. hominis* was recorded in 76.4% of municipalities, with predominance in the Southeast region, followed by the Midwest and South regions. In that survey, some municipalities in the North and Northeast regions were free of it. Farms located at altitudes between 400 and 1500 m are more affected, as are those with moderate to abundant rainfall and dense vegetation [69].

*Population dynamics.* The seasonality of *D. hominis* is determined by temperature and rainfall, that influences the emergence rate of pupae on the soil and the duration of the non-parasitic stages. These factors lead to infestation peaks during the rainy and warm season, from October to February, in the southern, southeastern, and midwestern regions of Brazil [70]. During this period, more than 80% of the total larvae recovered from cattle throughout the year may be found. However, the distribution of *D. hominis* larvae incidence in cattle throughout the months may not be repeated over the years [71].

### 6.4. Epidemiology of Cochliomyia hominivorax

Geographic distribution. Although the New World screwworm caused by *C. hominivorax* is widespread throughout the country, information on the distribution and epidemiology of this species in Brazil is scattered. The occurrence of myiasis was recorded in 96.2% of the municipalities from all Brazilian states in 1983. Such myiasis was the most prevalent cattle ectoparasitosis in the northeast states, but the highest number of records (87.6%) was in the southeast (34.2%), Midwest (30.8%), and south (22.6%) regions [72]. More recently, a comprehensive review of the occurrence of *C. hominivorax* in Brazil confirmed its wide distribution in the country, being recorded in 208 municipalities from all major Brazilian regions and biomes. In this survey, recorded cases of myiasis in livestock, wildlife, pets, and humans highlight the importance of this parasite species in a One Health context [73].

Population dynamics. In Brazil, New World screwworm in cattle is highly prevalent in the navel of newborn calves, secondary to routine handling practices that create wounds in the skin of the animals, and is also associated with very high tick burdens, that generate skin lesions [74]. Aggravated by favorable environments, this myiasis stands out among the main causes of calf mortality in some regions. In tropical areas, where temperature is more stable throughout the year, the birth season affects infestation occurrence, with a higher frequency in rainy periods [28]. The birth season with a higher probability of *C. hominivorax* larvae infestations in calves in the state of Rondônia was between October and April [75]. In late spring/summer, flies can be so numerous that common practices such as castration and dehorning are often delayed until the fly population is reduced by colder temperatures and low relative humidity. When rainfall occurs during all 12 months of the year, the incidence of the parasite tends to be constant throughout the year [76].

### 6.5. Control of Haematobia irritans, Dermatobia hominis, and Cochliomyia hominivorax in Brazil

#### Chemical Control

In Brazil, fly control relies on commercial insecticide products, most of which are neurotoxic to the insects and act on adult flies, and a few are IGRs acting on larvae. Currently, the Brazilian market for veterinary licensed ectoparasiticides comprises 301 products, categorized into six classes, with nearly half of these being mixtures of different classes of insecticides (Table 1).

SPs. The Brazilian market has 63 registered products for horn fly control containing SPs, of which 24 products contain only this insecticide class claimed for horn flies, and 39 products are mixtures, most with OPs and/or PBO. SPs are present in 57.3% of the insecticide products registered for horn flies. Cypermethrin is the most common active ingredient from this class, present in 54.5% of the products. Other pyrethroid active ingredients include cyfluthrin, alpha-cypermethrin, and flumethrin. All SPs have a pour-on or spray formulation. SPs also show therapeutic efficacy against *D. hominis*, however, without residual effects [77].

MLs. Currently, this is the second most represented class among registered insecticide products for horn flies in the country. Out of a total of 25 products, 22 exclusively have MLs as the active ingredient, while five products are mixtures. Insecticide products in this class are either pour-on or injectable, with a similar number of products in both cases. This is the main class for controlling *D. hominis* in Brazil, as it reaches 100% therapeutic efficacy [76,78] and a long residual period, allowing animals to remain free of infestation for more than 90 days after treatment [78], except for moxidectin (MOX), which does not have any registered claim against this parasite.

OPs. Only seven commercial products in the Brazilian market are based exclusively on active insecticides of this class. Active ingredients such as chlorpyrifos, coumaphos, diazinon, dichlorvos, ethion, fenthion, fenitrothion, and trichlorfon are present in 44 types of different commercial mixtures. By far, the most common OP is chlorpyrifos, which is present exclusively in mixed products (*n* = 30). The main OP formulations are pour-on and spray, but some ear tags of this class are commercially available on the national market. Like SPs, OPs have high therapeutic efficacy but no residual efficacy against *D. hominis* [77].

PPs. This class comprises nine products registered for horn fly control in the country. All of these contain fipronil as the insecticidal active ingredient, and about half of the commercial products contain only this insecticide as active ingredient. In contrast, the other products are pour-on mixtures with fluazuron (which has no efficacy against the horn fly) or an ear tag with diazinon.

IGRs. There are three IGR-based products on the Brazilian market. Two of them have diflubenzuron as their single active ingredient, administered orally in a mixture with sodium chloride, and are recommended for all animal categories in the herd, including calves. The third product is an injectable mixture containing novaluron and eprinomectin.

Isoxazolines. Recently launched in Brazil to control parasites that affect livestock, the only commercially available active ingredient of this insecticide class for bovines is fluralaner. This isoxazoline is claimed to control single and multi-host ticks, New World screwworm, horn flies and *Dermatobia.* The efficacy of a pour-on fluralaner treatment against *D. hominis* larvae was 98% at 3 days post-treatment (DPT), while persistent efficacy (>90% effectiveness) was found to last for up to 70 DPT. Treated animals remained free of larvae from 7 to 49 DPT [79]. Umifoxolaner is a novel second-generation isoxazoline ectoparasiticide developed specifically for use in cattle. Compared to first-generation compounds such as fluralaner, Umifoxolaner is designed to provide extended systemic persistence, which is expected to result in a longer duration of efficacy against key cattle ectoparasites, including *D. hominis* [79].

Insecticide mixtures. There are 48 insecticide commercial mixtures available, representing 43.6% of the insecticide products on the market for controlling horn flies. The majority are SP + OP mixtures (36.4%), divided equally between those with and without PBO. Less represented mixtures are SP + PBO (2.7%), ML + PP (1.8%), OP + OP (0.9%), OP + PP (0.9%), and ML + IGR (0.9%). In the treatment of *D. hominis*, the fipronil plus fluazuron mixture showed efficacy levels greater than 90% from 7 to 35 days post-treatment, reaching 99.5% efficacy after 21 and 28 DPT [80]. A pour-on mixed formulation with cypermethrin, chlorpyrifos, PBO, and fluazuron showed therapeutic efficacy against *D. hominis* [81].

Insecticide products for horn fly control are mainly administered as pour-on (60.9%) and spray commercial formulations (20.9%), with other administration routes including injectables (11.8%), ear tags application (4.5%), and oral routes (1.8%).

### 6.6. Strategies for the Control of Flies on Cattle in Brazil

The conventional control of horn flies involves tactical or strategic treatments based on the need for immediate action or the seasonality of the fly, respectively. Tactical control is theoretically based on the economic threshold for the species. However, due to the enormous difficulty of making decisions based on counting large numbers of flies, in practice, chemical treatments are often carried out by taking advantage of livestock management or through empirical decisions, frequently executed abusively and erroneously. The conventional strategic treatment of horn flies targets population peaks associated with the beginning and end of the rainy season, typically using spray or pour-on formulations. However, the long interval for the peak to occur (several months) and its short duration (a few weeks) [62] contrast with the increasingly shorter protection of these formulations, jeopardizing the timing and effectiveness of such control.

An appropriate recommendation for horn fly control may consider the “strategic planning” of treatments (targeting the likely period of the highest level of infestations) and a “tactical execution” (waiting for the adequate moment, if necessary). The observation of herd behavior may be a helpful clue in decision-making.

Recent studies have shown that infestations do not significantly affect the weight gain of Nelore cattle up to 18 months of age, provided there is a good pasture supply. However, such an impact became evident in the first dry season and the following rainy season from this age onwards [9]. In principle, the strategic treatment of horn flies should target animals from 18 months of age onwards and/or be carried out tactically when infestations and herd behavior indicate a real need.

To delay resistance problems, it is recommended to rotate insecticides with different modes of action, which in practice means alternating the insecticide class of the product in most cases, as well as avoiding products from insecticide classes that have not demonstrated proper efficacy. Although it may impact on a higher cost of treatment, it is essential to consider that the greater the variety of insecticide classes used successively in the rotation adopted on the property, the lower the chances of resistance and control problems and the better the results in terms of efficiency, investment, and operational effort.

## 7. The Best Management Practices for the Sustainable Control of Flies That Affect Livestock in Colombia

### 7.1. An Overview of Cattle Demographics in Colombia

Livestock farming is a key sector in Colombia’s economy, characterized by its diversity and adaptation to different geographic regions. Colombia is one of the leading beef producers in LA, after Brazil, Argentina, and Mexico. According to ICA [82], cattle herds are mainly concentrated in the departments of Antioquia (11%), Meta (8%), Casanare (8%), Córdoba (8%), Caquetá (7%), Bolívar (6%), Cesar (6%), Magdalena (5%), Santander (5%) and Cundinamarca (5%), which represent 75% of the total population. The bovine population in the country is distributed across 638,941 properties and totals 29,194,104 animals, representing a 1.5% reduction compared to 2023 [83]. Each area has distinct characteristics that influence the type of livestock and the corresponding management practices. For beef production, breeds such as Brahman and zebu are specifically adapted to tropical climates.

The livestock production structure in Colombia is highly heterogeneous, although improvements have been made in new breed types, pastures, and nutrient management, among other areas. Production is characterized according to the following structure: extensive (6.2%), traditional extensive grazing (61.4%), improved extensive grazing (28.4%), enhanced intensive grazing (3.5%), and confinement (less than 1%) [84].

Livestock farming is one of the most significant economic activities in Colombia, occupying approximately 30% of the national territory and generating both direct and indirect jobs. About 7000 million liters of milk and 8,000,000 tons of meat are produced annually. However, it also is negatively impacted by some environmental challenges, such as soil degradation, inefficient use of natural resources, and the effects of climate change [85].

In Colombia, economic losses associated with fly infestations have been superficially estimated and reported primarily in livestock-oriented publications. Excessive fly burdens in production systems have been associated with estimated losses of approximately COP 172,500 (~USD 43) per individual beef cattle and COP 448,500 (USD 112) per dairy cow. These values highlight the need for a deeper understanding of the impact of these insects on farm productivity and the development of effective management strategies [86].

### 7.2. Epidemiology of Haematobia irritans

Geographic distribution. The horn fly is ubiquitous in Colombia, found from sea level to 4000 m above sea level. It is found mainly in the tropical and subtropical regions of Colombia, where temperatures and humidity are conducive to its development. This includes areas of the Eastern Llanos, the Caribbean region, and some areas of the Coffee production Axis. A survey conducted in 1984 revealed that 51.1% of cattle examined in the Llanos of Colombia were infested with this fly [87].

Population dynamics. The largest populations of horn flies occur at the beginning and end of the rainy season, respectively, in April and May and between September and December. Benavides et al. [88] stated that there is association between rainfall and parasite load. At the beginning of the second or third quarter of the year, the most critical population peak generally occurs, with decreases following the intensity of the dry season each year, which serves as a population regulator. This could lead to the formulation of climate-based treatment regimens. From the use of conventional insecticides, such as IVM, to the mixture of cypermethrin and chlorpyrifos and from natural alternatives, including plant extracts, to innovative silvopastoral systems, the need for diverse and sustainable approaches to the management of horn flies has been highlighted. Although these studies have been valuable, most of them date back more than a decade, which highlights the urgency to continue researching this parasite and its control methods to adapt them to current conditions and improve their efficacy in infestation management [88].

### 7.3. Epidemiology of Dermatobia hominis

Population dynamics. There is no information on the population dynamics of this fly in Colombia. It is known that this furuncular myiasis in cattle primarily occurs in dark-colored cattle in the coffee-growing region.

Geographic distribution. Unfortunately, studies on this parasite in Colombia are limited. Cases of *D. hominis* in Colombia have been reported in the states Antioquia, Amazonas, Cundinamarca, Eje Cafetero, Tolima, Nariño, Norte de Santander, Meta, and Choco [89].

### 7.4. Epidemiology of Cochliomyia hominivorax

Geographic distribution. Little is known about this parasite in Colombia. To date, no published studies are available on its geographic distribution across the different climatic zones and agroecological areas, the annual frequency of positive cases in domestic animals and humans, or the economic losses in Colombia. A study in Puerto Boyacá in 2009 showed the very low prevalence of myiasis due to *C. hominivorax* (0.4%) among the 12,325 cattle evaluated. However, it affected 41% of the 44 livestock farms selected in Puerto Boyacá. Cattle age was associated with the presence of myiasis caused by *C. hominivorax*, indicating that younger cattle have a higher risk of infestation compared to adults. This could be associated with newborns’ navels natural exposure, which increases the risk of myiasis occurrence [90]. Inappropriate and ineffective products such as creolin, gasoline, and burnt oil are used in the treatment of traumatic myiasis in cattle, which may aggravate even more the disease. ICA (Colombian Agricultural Institute) data in the Antioquia Uraba region showed cases of myiasis in cattle and pigs caused by *C. hominivorax*, in an area with rainfall ranging from 1800 to 4000 mm and temperatures between 22 and 29 °C [90].

At a Hospital in Medellin, Urbano del Valle et al. [91] found that most cases of myiasis in humans occur during the warm and humid times of the year, mainly from November to February.

### 7.5. Chemical Control of Haematobia irritans, Dermatobia hominis, and Cochliomyia hominivorax in Colombia

#### 7.5.1. Chemical Control

The use of insecticides is regulated by various laws and resolutions that seek to protect public health and the environment. The Colombian Agricultural Institute (ICA) is the entity responsible for the registration and control of chemical pesticides for agricultural use. According to ICA data (2016–2018), Colombia is a significant pesticide user—ranking 18th globally—with herbicides making up ~50% of total sales.

The insecticides used to control *H. irritans*, *D. hominis* and *C. hominivorax* in cattle from Colombia are presented in Table 1. The control of bovine infesting flies in Colombia frequently relies on chemical interventions, among which endectocides such as IVM are prominent, typically administered via subcutaneous injection to achieve systemic activity against a broad spectrum of parasites. There are about 200 products available for bovine use in Colombia that contain IVM as the main active ingredient [83].

IGRs, including diflubenzuron, are employed either as oral boluses or incorporated into feed supplements to disrupt the chitin synthesis pathway of the insects, thereby inhibiting the development of larval stages. Despite their demonstrated efficacy, the sustained and repetitive use of these compounds, particularly without rotation among active ingredients with differing modes of action, has accelerated the development of insecticide resistance in fly populations. This phenomenon undermines long-term control efficacy and demands the adoption of integrated pest management frameworks that integrate biological, cultural, and environmental control measures to ensure the sustainable suppression of vector and pest populations [92].

The treatment of *C. hominivorax* includes the mechanical removal of larvae if possible, thorough wound debridement, and the use of larvicidal agents (e.g., IVM), in addition to topical repellents in spray formulations. No scientific publications have reported chemical resistance of this parasite in Colombia. Reduction in the efficacy of MLs has been suggested in the field and subsequent case evaluations have revealed inappropriate product use and the administration of unlicensed drugs; therefore, resistance has not been conclusively demonstrated so far.

For dairy cattle, chemical products like topical adulticides (OPs and SPs, either alone or in mixture) are applied in pour-on formulations and spray. Additionally, IGR larvicides (like diflubenzuron) are given orally to break the larval cycle in the manure of *H. irritans* in dairy cattle. To avoid resistance and guarantee long-term management, chemicals must be mixed with biological and management strategies.

Cattle flies, especially *H. irritans* in Colombia, are becoming more resistant to widely used pesticides. Because of their initial success and ease of use, pyrethroid insecticides (such as cypermethrin) have been used extensively; nevertheless, research suggests that their effectiveness is declining because of resistant populations. Although country-specific resistance data remain limited, it is well established that the intensive and repetitive use of the same insecticides creates selective pressure, allowing resistance genes to persist at high prevalence for years, even after the product is withdrawn from use [29]. Regional research and worldwide experience highlight the urgent need to implement rotation of insecticides pertaining to different chemical classes and IPM programs and diversify control measures to ensure sustainable and successful fly management, even though there is currently a lack of direct evidence of resistance on these pest dipterans within Colombia.

#### 7.5.2. Strategies for the Control of Flies on Cattle in Colombia

One of the most common approaches in Colombia is the use of IVM to control *H. irritans* mainly in the rainy season. This treatment helps to reduce the parasite load on cattle, thereby improving their overall health and productivity. Another conventional treatment that has been investigated in the country is the mixture of cypermethrin and chlorpyrifos. This proved highly effective in controlling natural infestations of *H. irritans* in Holstein cattle in Antioquia. This treatment achieved a 99.5% control rate at 21 DPT, which indicates its effectiveness, particularly in instances where resistance to other products has emerged [93].

The control of *D. hominis* is primarily based on the use of OPs, followed by IVM or DRM (subcutaneous). The formulation of cypermethrin 15% + chlorpyrifos 25%, applied as pour-on on cattle infested with *D. hominis* larvae, showed an efficacy of 82.7–96.3% [94]. Direct control methods also include the manual extraction of *D. hominis* larvae out of the skin through manual squeezing.

## 8. The Best Management Practices for the Sustainable Control of Flies That Affect Livestock in Mexico

### 8.1. An Overview of Cattle Demographics in Mexico

Cattle graze on nearly 2 million square kilometers of land in Mexico, which is divided into four pastoral regions based on agroclimatic conditions (arid and semiarid, temperate, dry tropical, and humid tropical). The climate of each region has a significant impact on production systems. According to estimates, there are 32.40 million cattle in the country (2.42 million dairy and 29.99 million beef) [27]. Calves (7.29 million), steers/heifers (7.78 million), and cows (1.73 million dairy and 16.60 million beef and dual use cows) account for the age-based integration of cattle production.

Extensive grazing, controlled grazing, restricted production, and semi-confined production are the mainstays of the Mexican cattle production system. Open-range (or open grazing) techniques are used to raise 55% of all cattle in the country. The production of beef cattle is usually linked to open grazing, which is more common in the northern states. Nineteen percent of cattle are produced by controlled grazing. Confinement is typically utilized in beef feedlots (13%) and dairy production. Some dairy and dual-purpose agriculture (7%) uses semi-confinement. Cows for export, local beef production, and dual-purpose beef and dairy production are Mexico’s three primary commercial beef cow production activities [95].

### 8.2. Epidemiology of Haematobia irritans

Geographic distribution. As of right now, *H. irritans* is known to be found all over the nation, mostly in large systems that support its life cycle in conjunction with livestock. *H. irritans* is found in the temperate, tropical, and subtropical regions of Mexico [8].

Population dynamics. *H. irritans* population dynamics typically show bimodal behavior with significant intra-annual changes. Temperature and relative humidity are linked to infestation seasonality, and the infestation index decreases at high elevations and changes more in tropical than temperate locations. *H. irritans* infection rates are highest from late spring to early autumn, with up to three population peaks in some regions [29].

**Figure 5:**
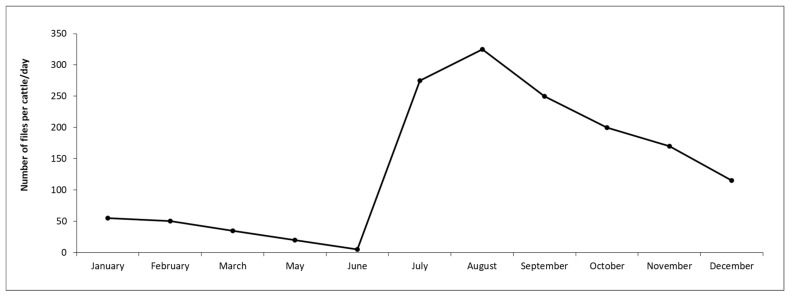
Population dynamics of *Haematobia irritans* on cattle in southern Mexico [96].

Rainfall and humidity are linked to *H. irritans* infestations on cattle in the tropical parts of Mexico. Figure 5 [96] shows the annual population dynamics of *H. irritans* on cattle in southern Mexico.

*H. irritans* infestations on cattle can reach over 4000 flies per animal in northern Mexico throughout the summer. In contrast, infestations can decrease to 200–450 flies per animal during unfavorable times. *H. irritans* population dynamics are bimodal and seasonal in temperate areas, peaking in infestation rates in the summer and increasing from late spring to early autumn. Animal infestations are not found in temperate climates because facultative diapause may occur during the winter [97].

In the Mexican tropics, *H. irritans* often infests cattle between June and December [29]. Between August and November, cattle in the hot, humid tropical state of Veracruz are heavily infested with *H. irritans* (70 to 121 flies/animal) [98]. According to Galindo-Velasco et al. [99], cattle in the state of Colima exhibited high *H. irritans* infestations (120 to 236 flies per animal) during six months of the year (June to November), considering sub-humid tropical and subtropic environments. However, throughout the year, cattle in the state of Tamaulipas were found to have significant levels of horn fly infestation, with population peaks surpassing 200 flies per animal found in April, May, June, and September [100]. It is unknown how many generations *H. irritans* produces annually in Mexico.

### 8.3. Epidemiology of Dermatobia hominis

Geographic distribution. In Mexico, *D. hominis* has been reported in humans, cattle, and other vertebrates, mainly in the southeast and north of the country [13]. In a study conducted in Chiapas, in a rainy and mountainous jungle region, Hernández-Jiménez et al. [13] found that 10.6% of the animals had nodules on their skin and subcutaneous tissue produced by *D. hominis*. Likewise, they found between 1 and 22 nodules per cattle, distributed in four body regions (tail, leg, abdomen, and scapula).

Population dynamics. The natural habitat of *D. hominis* consists of jungle areas, with a warm, humid climate and low altitude, which is more common in areas around 600 m above sea level. It is rarely observed above 1400 m above sea level [13]. In the Yucatan Peninsula, the prevalence of furuncular myiasis due to *D. hominis* increases at the beginning of the wet season, reaching a peak in October [101]. In the case of cattle, zebu breeds are the least infested [102].

### 8.4. Epidemiology of Cochliomyia hominivorax

Geographic distribution. *C. hominivorax*, the New World screwworm agent, was eradicated from Mexico in 1991 through a transnational federal program that included the intensive and massive release of sterile adult male flies and other direct control measures applied to animal wounds. However, since November 2024, the reintroduction of this fly species has been reported in Chiapas, Oaxaca, Tabasco, Puebla, Veracruz, Quintana Roo, Campeche, Yucatán, Guerrero, Querétaro, Nuevo León, Morelos, and Jalisco (Appendix B) [103,104].

### 8.5. Chemical Control of Haematobia irritans, Dermatobia hominis, and Cochliomyia hominivorax in Mexico

The insecticides used to control *H. irritans*, *D. hominis*, and *C. hominivorax* in cattle from Mexico are presented in Table 1. The prominent families of insecticides used to control these dipteran flies in Mexico and their uses, efficacy, and persistence are detailed below:

OPs. The most used OP compounds are coumaphos at 20% (spray), diazinon ear tags at 21.4%, and ethion at 15%, all of which are generally used to control pyrethroid-resistant populations of *H. irritans*. Another insecticide-impregnated ear tag, containing diazinon 30% and chlorpyrifos 10%, is used to control *H. irritans*. Diazinon 21.4% ear tags provided greater than 87% control of *H. irritans* for 90 days [105]. For the local treatment of New World screwworm infested skin lesions, the veterinary health authorities of Mexico recommend the use of coumaphos 3% with proporxur 2% topically.

SPs. Mainly cypermethrin (22.4%), deltamethrin (25%), and flumethrin (1–3%) are applied by pour-on and aspersion routes. In addition, a mixture of cypermethrin 15% + chlorpyriphos 25% and chlorpyriphos 24% + permethrin 5%, cypermethrin 5% + ethion 15%, and permethrin + PBO are used to control *H. irritans* [29]. López-Valencia et al. [95] evaluated the effectiveness of a mixture of cypermethrin 15% and chlorpyrifos 25% in a pour-on solution against *D. hominis* larvae in cattle. On 3 DPT, a larvicidal efficacy of 98.3% was achieved, and by 28 DPT, the efficacy dropped to 82.7%.

PPs. A 1% fipronil-based pour-on formulation is used against *H. irritans* in cattle with >80% efficacy [29]. For local treatments of *C. hominivorax* larvae infestation, commercial topical fipronil spray application is recommended.

MLs. IVM-1%, IVM-3.15%, and IVM-4%; DRM-1%; and MOX-1% and MOX-10% (all administered subcutaneously) are used in Mexico to control nematodes, ticks, and flies on cattle. In grazing cattle in Tuxpan, Veracruz, injectable IVM has shown an efficacy of over 90% in reducing horn flies for up to 90 days after treatment [105]. A mixture of IVM (0.45 mg/kg) + abamectin (0.25 mg/kg) is used to control ticks, nematodes, and flies of cattle in Mexico. For the control of *D. hominis* larvae, the use of IVM injectables (subcutaneously 0.2 mg/kg BW) is recommended [103]. With the reintroduction of New World screwworm in Mexico, the use of injectable IVM for its treatment and prevention is recommended by government veterinary health authorities.

IGRs. Oral diflubenzuron is used (1 g/animal/day) with good results for controlling *H. irritans* larvae development in cattle feces [29].

Isoxazolines. Recently launched in Mexico to control ectoparasites that affect livestock, fluralaner is the only commercially available active ingredient of this insecticide class. However, its efficacy in controlling cattle parasitic flies is not known to have been tested in Mexico.

### 8.6. Strategies for the Control of Flies on Cattle in Mexico

In the Mexican tropics, cows graze longer at night to protect themselves from high temperatures and sunlight incidence. Due to heavy fly infestations, changes in grazing behavior have been documented in some regions. In these cases, it has been observed that cows huddle together to reduce their exposed body surface to avoid the dipteran attacks [8].

In the tropics, strategic treatments are recommended mainly in the rainy season when *H. irritans* infestation can reach more than 200 flies per animal. Special attention should be paid to animals of European cattle breeds and bulls. In the Mexican tropics, young animals can also be parasitized by *H. irritans* but to a lesser extent than adult animals, especially bulls. The use of coumaphos by spray or ear tags containing diazinon or diazinon plus chlorpyrifos is recommended topically every two weeks (spray) and every three months (ear tags). Additionally, during the rainy season, the use of MLs (e.g., IVM, DRM, or MOX) is recommended to control ticks and nematodes. Young cattle only need to be treated with insecticides in the high-infestation season, once or twice a year [8].

*D. hominis* is common in southern Mexico, primarily in Chiapas’s rainy and forested mountains [13]. In this area, it is recommended that infestations be prevented during the rainy season by administering MLs (e.g., IVM, DRM) every two months. In clinical cases, it is advisable to apply coumaphos 3% with proporxur 2% topically directly to the nodules and administer IVM-1% or DRM-1% subcutaneously (0.2 mg/kg body weight) [13].

Regarding *C. hominivorax*, the National Service for Agri-Food Health, Safety, and Quality (SENASICA) has developed a prevention plan based on defensive barriers that reinforce preventive measures on the southern border of Mexico. When maggot infestations are detected in an animal shipment and confirmed to be caused by *C. hominivorax* larvae at Federal Internal Verification Points (FIPs), one of the main actions implemented is the topical treatment of infestations with larvicidal products (e.g., coumaphos and propoxur). In addition, all cattle in the shipment are administered IVM-1% intramuscularly, treated with chlorpyrifos, and monitored under official surveillance.

## 9. The Best Management Practices for the Sustainable Control of Flies That Affect Livestock in Uruguay

### 9.1. An Overview of Cattle Demographics in Uruguay

Uruguay has 16.4 million hectares dedicated to agricultural use, which corresponds to more than 90% of its total territory. Forty percent of such rural properties are related to livestock production and 30% to agricultural production. The remaining 30% is divided between livestock (dairy), rice, and forestry [106].

The primary method of beef production is the raising of free-range animals in natural fields, which occupy 79% of the area dedicated to grazing. The area of improved pastures is 19%, with the remaining 2% being grazed on forested areas. Only 15% of the animals are raised for fattening in pens. The cattle population is approximately 12 million animals, the predominant breeds being Hereford and Aberdeen Angus, and 80% of the beef produced is exported [106].

In recent years, national research in cattle has primarily focused on *H. irritans* and *C. hominivorax*. *D. hominis* may represent a higher incidence in some regions of the country. The damage to public health corresponds to the myiasis produced by the larvae of *C. hominivorax* and *D. hominis* in humans.

### 9.2. Epidemiology of Haematobia irritans

Geographic distribution. *H. irritans* has been present in Uruguay since 1991 and has gradually dispersed towards the south of the territory [107]. It is currently distributed throughout the country between parallels 30° and 35° south latitude.

Population dynamics. Different groups of researchers carried out epidemiological studies in both beef and dairy cattle, in the north and south of the country.

**Figure 6:**
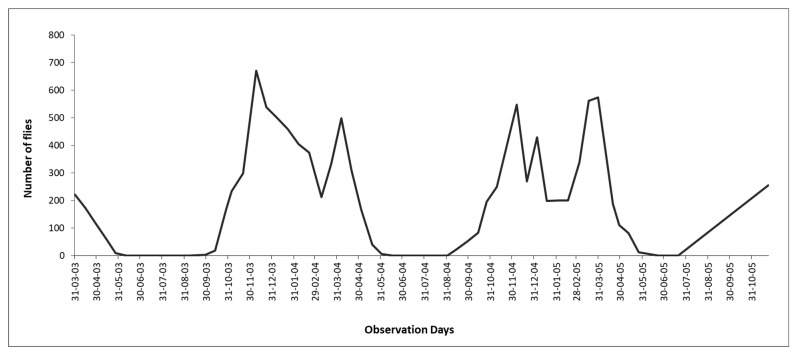
Population dynamics of *Haematobia irritans* dairy cattle in Uruguay [109].

Given the temperate climate conditions, *H. irritans* presents bimodal behavior in Uruguay. A population dynamic study was conducted in the Department of San José (34°15′ south latitude, 56°5′ west longitude) from 2003 to 2005. The results confirm, once again, the bimodal behavior of the fly population during the year with a peak in autumn at the beginning of April and another in spring at the beginning of December (Figure 6). For two consecutive winters, no adult flies were found on animals, so the spring populations were likely to be due to individuals that emerged from pupae that were in diapause [108].

The average number of flies per animal in the peaks was 620 in the spring and 450 in the fall for the first fly season. The peaks for the second were similar (548 and 574, respectively).

Although the period of the highest infestation occurred twice a year, for 90 continuous days in spring and 60 days in autumn (Figure 6), the animals supported loads equal to or greater than 200 flies per animal without significant economic losses. During the summer, the population decreased, but it remained close to 200 flies/animal. Contrary to what was observed in beef cattle on extensive farms, the horn fly population in dairy cows was significantly higher [109].

In all studies, it was observed that the favorable season for horn fly development begins in October and ends with the first frosts in April. The trend in fly population variation in these areas is bimodal, with peaks towards the end of spring and the beginning of autumn. During the winter of 2000, no fly records were registered. In the second winter of 2001, with an average temperature slightly lower than 15 °C, the presence of flies was confirmed at a very low number. It can be inferred that the maintenance of populations during the winter may be due mainly to pupae that enter diapause or to the presence of some remaining adult individuals [110].

The most critical factors for the development of *H. irritans* (from egg to adult) were temperature and rainfall. Both larvae and pupae die when they are immersed in water for periods greater than 6 h. Between 12 and 15 generations can develop yearly, mainly from October to April [111].

### 9.3. Epidemiology of Cochliomyia hominivorax

Geographic distribution. Cases of New World screwworm have been reported throughout the territory between parallels 30° and 35° south latitude [112]. An epidemiological survey of 382 livestock producers concluded that all establishments had myiasis problems, with prevalences of 4.5% (1.3–9.5%) in cattle and 6.2% (2.5–14.6%) in sheep, varying according to the areas. A lethality of 4.5 and 18.5% in cattle and sheep was registered, respectively [113].

Population dynamics. Uruguay is situated in a transitional zone where the *C. hominivorax* fly exhibits seasonal behavior, primarily from spring to autumn. North of parallel 32° south latitude, it can be presented all year round [114]. The survival of pupae in winter is closely tied to the average temperature of the environment. Marques et al. [114] investigated the survival of the pupal stage under adverse climatic conditions in Uruguay in 2016 and 2017. They found that the reproductive capacity of adults emerged when the pupal period was equal to or greater than 15 days.

In winters with favorable climatic conditions in northern Uruguay (Artigas and Tacuarembó), pupae survive, and the adults that emerge maintain their reproductive capacity. However, in unfavorable winters with lower temperatures in southern Uruguay (Cerro Colorado—Florida and Montevideo), the emergence of adults is interrupted. These results suggest that fly populations, and consequently myiasis, decrease substantially in winter due to unfavorable weather conditions. In Uruguay, with a monthly average temperature of 12.6 °C, the non-emergence of adults occurs. At an average temperature of 15.7 °C, adults begin to emerge [114].

The average environmental temperature also influences the duration of the pupal period. The higher the temperature, the shorter the pupal period. As winter progresses and the environmental temperature decreases, pupal periods become longer. Due to this association, pupal periods are shorter in the north than in the south of Uruguay. To the south of the Negro River, the most extended pupal period (57 days) was recorded, with an emergence percentage of adult flies of 0.5% [114].

Additionally, in a forest ecosystem in Uruguay, the survival of L3 and pupae of *C. hominivorax* was studied during May, June, and July. Adults began to emerge after 7–10 days (14–15 °C, relative humidity 88–97%), with pupal survival ranging from 42 to 47 days [109].

### 9.4. Control of Haematobia irritans and Cochliomyia hominivorax in Uruguay

Chemical control. The insecticides used to control *H. irritans* and *C. hominivorax* in cattle from Uruguay are presented in Table 1.

The most used insecticidal products in Uruguay are based on SPs, OPs, and fipronil. On a smaller scale, MLs are used, and currently, isoxazolines are also being used. The administration routes used in Uruguay to control *C. hominivorax* include topical (powder, aerosol, paste, liquid), injectable, and pour-on formulations.

In Uruguay, depending on the production systems and the fly populations present, there are two scenarios of *H. irritans* control:

Castro et al. [115] demonstrated that fly populations in Uruguay are not always large enough to affect weight gain in animals managed under extensive production systems. For a negative effect on weight gain in beef cattle grazing natural pastures to become evident, it is likely not only that high fly populations are required but also that these populations persist for prolonged periods. According to Miraballes et al. [7], selective treatment strategies focusing on bulls and the 20% of cows with the highest parasite burden proved to be highly effective for controlling *H. irritans* populations.

In dairy cattle, a walk-through fly-trap can be used, which has been shown to reduce the number of flies on animals by more than 80% [7].

In livestock with high productive demands (such as milking cattle, supplemented fattening, and high production), *H. irritans* can cause significant losses, making it necessary to apply a rational control method.

In livestock with high productive demands, the strategic objective is to avoid the presence of large populations of *H. irritans* in both spring and autumn while maintaining infestation levels below the threshold of 200–250 flies.

**Figure 7:**
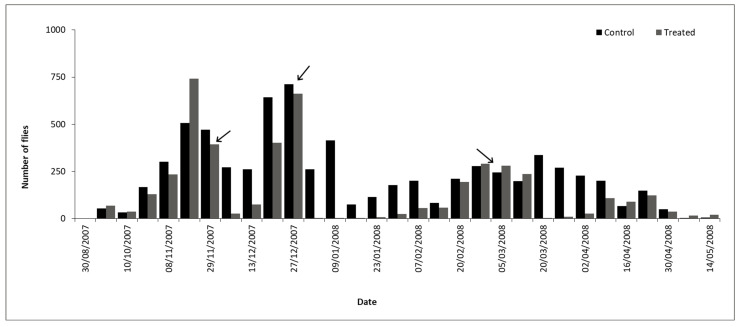
Strategic treatments (arrows) for the control of *Haematobia irritans* in Uruguay [116].

A field trial conducted in Lavalleja, Uruguay, using Hereford cattle evaluated a selective fly control strategy during one fly season, based exclusively on products with zero withdrawal time for meat and milk. The sequential applications of pirimiphos-methyl and cypermethrin + PBO significantly reduced fly burdens in treated animals compared with controls. Treated cattle showed a mean reduction in fly load of 34% during spring and 41% during autumn (Figure 7). All products were fully effective immediately after application and exhibited the expected residual activity [116]. In Uruguay, treatments with pirimiphos-methyl had high efficacy and residuality in line with what was expected for this formulation, as well as cypermethrin + PBO, which coincided with the previous diagnosis of metabolic resistance [116].

Treatment against *C. hominivorax* is based on the use of curative and prophylactic products. In clinical cases of animals with myiasis, it is recommended first to apply a topical insecticide registered in the Ministry of Livestock, Agriculture and Fisheries (MGAP) of Uruguay to kill *C. hominivorax* and then extract mechanically the larvae. Live larvae must be prevented from falling to the ground to burrow and pupate. The larvae can be treated with insecticide or transferred to a container for later destruction. The treated animals must be kept in observation, depending on the extent of the lesion; they generally must be treated on more than one occasion because the lesions caused by *C. hominivorax* larvae can become very deep and slow to heal both due to the bacterial contamination they present and because the dead larvae in the wound act as a foreign body.

## 10. Resistance Status of *H. irritans*, *D. hominis*, and *C. hominivorax* to Insecticides in LA

*H. irritans.* In Argentina, the resistance of *H. irritans* to cypermethrin was determined for the first time in 1997 in a property where flies had shown susceptibility to this drug since 1993 [40]. Another study carried out between 1999 and 2000 in 85 establishments in northern and central Argentina indicated the presence of the resistance of *H. irritans* to SPs in more than 95% of farms [40].

In Brazil, the resistance of *H. irritans* to insecticides has been reported so far only for SPs [9], a situation that has been widespread in the country for more than two decades. Horn fly resistance to pyrethroids is mainly caused by the enhanced activity of P450 mono-oxygenases and, secondarily, by the reduced sensitivity of the target site (knockdown resistance—*kdr*), as detected in the state of Mato Grosso do Sul [117]. The *kdr* mutation was detected at relatively low frequencies in about half of the pyrethroid-resistant populations from all Brazilian regions except the Northeast [118]. Also, an esterase-mediated mechanism of resistance was found in horn fly populations [117]. Although resistance to OPs has not yet been officially reported, strong evidence of the reduced efficacy of OP products in field studies [9] and several reports from cattle producers and industry personnel over the years strongly suggests that OP resistance is very likely to occur in the country and that its official reporting depends only on a proper diagnostic. So far, no suspicion of horn fly resistance exists for other insecticide classes in Brazil.

In Mexico, the resistance of *H. irritans* to SPs (e.g., cypermethrin and permethrin) was documented in the late 1990s initially in states such as Tamaulipas, Veracruz, and San Luis Potosi, then in Nuevo Leon, Chiapas, Tabasco, Jalisco, Sinaloa, Colima, and Yucatan [8]. Low resistance to diazinon in northern Veracruz and Nuevo León has been reported. In Guerrero, resistance to both OPs and SPs was found in 100% of 30 sampled farms [29].

In Uruguay, populations of *H. irritans* resistant to SPs (cypermethrin) have been reported, associated with mutations in the *kdr* and super-*kdr* genes, as well as metabolic resistance [107]. To date, no cases of *H. irritans* resistant to insecticides have been reported in Colombia.

*D. hominis.* Failures in *D. hominis* control after the treatment of cattle with MLs have been reported in Brazil. On two farms in the southeastern region of Brazil, with a history of the continuous use of MLs, with three and six applications per year in each, IVM showed no or reduced efficacy 7–10 days after treatment [119]. In a randomized, double-blind, controlled field study performed in a farm in the midwestern region, injectable DRM showed a maximum efficacy of 86.3% on the 14th DPT; after treatment, 90% of the animals remained parasitized with live larvae on the 7th DPT and 70% on the 14th DPT, while in the control group, all animals were infested on all dates [120]. To date, no cases of *D. hominis* resistant to insecticides have been reported in Argentina, Colombia, Mexico, or Uruguay.

*C. hominivorax.* In a study conducted in Argentina, Anziani & Suarez [45] demonstrated that DRM (0.2 mg/kg bw) was less than 60% effective in preventing myiasis post-castration by *C. hominivorax* in cattle. In this study, third-stage larvae in the 10 days following treatment were able to pupate and emerge as adults. Recently, Muchiut et al. [51] reported that DRM-1% and IVM-3.15% did not prevent the development of scrotal myiasis in most castrated calves in a facility in the north of the province of Santa Fe.

In Brazil, the insecticide resistance of the NWS to OPs and MLs is well known. Carboxylesterase mutations associated with OP resistance occur in *C. hominivorax* populations from different regions of the country [121,122]. Also, a mutation associated with pyrethroid hydrolysis has been detected in samples from Brazil and Uruguay, Paraguay, Venezuela, Colombia, and Cuba [123]. The ineffectiveness of ML treatments in the prevention and treatment of myiasis in cattle has been a frequent complaint from ranchers in Brazil. Such complaints were confirmed by the ineffectiveness of ivermectin (pour-on and subcutaneous) and abamectin (pour-on, subcutaneous, and intramuscular) applications in preventing scrotal myiasis in castrated cattle in southeastern Brazil [124].

## 11. Non-Chemical Control of *H. irritans*, *D. hominis* and *C. hominivorax* in LA

### 11.1. Non-Chemical Control of Haematobia irritans

Physical control. Physical control of *H. irritans* involves the use of walk-through traps, where cattle pass through a darker corridor and the flies are removed by strips, being attracted to the lighter areas on the trap sides and remaining trapped there. Key aspects include the trap location and the cattle’s adaptation period to the corridor, which requires a gradual assembly process. In Uruguay, this technique showed a reduction of more than 82% in horn flies [125]. In Argentina, the traps have also been tested occasionally. The main advantage of this method is that the physical control of *H. irritans* can reduce insecticide use and the consequent selection of drug-resistant populations [8].

Biologic control. The natural enemies of *H. irritans* in LA include pteromalid parasitoids (i.e., *Muscidifurax* spp. and *Spalangia* spp.) that can parasitize fly pupae [126]. Entomopathogenic bacteria such as *Bacillus thuringiensis*; entomopathogenic nematodes like *Steinernema* spp. and *Heterorhabditis* spp.; and entomopathogenic fungi like *Beauveria* spp., *Metarhizium* spp., and *Isaria* spp. are also used to reduce fly populations [8].

Parasitoids attack the pupae of different fly species and are available in Mexico for use in livestock. These products are sold in cloth bags or plastic containers with housefly pupae parasitized by one or two wasp genera (*Muscidifurax* and/or *Spalangia*). A number of species have been reported, including *Spalangia endius*, *S. nigroaenea*, and *Muscidifurax raptor*, which are frequently found [126]. In the Pantanal biome of Brazil, about 10% of *H. irritans* pupae were found to be parasitized by *S. nigroaenea*. The data suggests a role in the natural control of this fly in the area [127].

In LA, different isolates of *Beauveria bassiana* (Bb), *Metarhizium anisopliae* (Ma), and *Isaria fumosorosea* have been tested in vitro for the control of adult *H. irritans*. A controlled study conducted in a dry area of Mexico tested different formulations on cattle. The data revealed that five *M. anisopliae* strains controlled more than 94% (up to 100%) of infestation after 12–13 DPT. In comparison, three *I. fumosorosea* strains decreased the generation of immature phases from 90 to 98% up to 13 DPT [8].

The efficacy of *M. anisopliae* was also evaluated to control the adult and larval stages of *H. irritans* in farms in Brazil. The ingestion of microencapsulated fungal spores by heifers significantly reduced (*p* > 0.05) horn fly emergence from dung pats. When treated with a spray of encapsulated conidia suspensions, it reduced the number of horn flies from 29.5 (control group) to 11.7 (treated group) [128].

An aqueous formulation of the strain Ma134 of *M. anisopliae* was tested in dairy cattle infested with *H. irritans* in semiarid Mexico. The compound reduced 68% of the infestation after 4 weeks [129]. Another Mexican strain Ma135 was tested against *H. irritans* in dairy cattle. The product used a mixture pasture/corral system, resulting in a 58% reduction in *H. irritans* infestation after 6 weeks [130]. The main disadvantage of using entomopathogenic fungi as a treatment is the possible deactivation of conidia by ultraviolet radiation. Moreover, fungi live in high-humidity environments that are difficult to monitor in many climatic regions. Therefore, the application of fungi must be used only before sunrise to sustain their growth and potential efficacy. Although these strategies have shown moderate efficacy in controlling *H. irritans*, they are not yet commercially available for veterinary use in LA.

In LA, dung beetles of the Scarabaeidae family can also play a crucial role in controlling *H. irritans*. Their activity involves degrading cattle feces and burying them in the soil, thereby preventing the development of the free-living phase of *H. irritans* [131]. A study conducted by Basto-Estrella et al. [132] in southeast Mexico found that dung beetles removed up to 40% of cattle manure, thereby reducing the *H. irritans* larval population.

In the late 1980s, the African dung beetle *Onthophagus gazella* (now known as *Digitonthophagus gazella*) was introduced to Brazil. After laboratory mass rearing, it was released nationwide in partnership with a commercial company to reduce the *H. irritans* population. Unfortunately, there are no data on the impact of this species on horn fly populations. However, some effects are expected after a period of species adaptation to the different environments and stabilization of beetle populations.

Silvopastoral system. An effective strategy for controlling this parasite in Colombia, Brazil, and other countries is the implementation of integrative silvopastoral systems. The strategy is to implement trees, forage shrubs, and pasture plots for cattle raising to diversify agroecosystems. The concept involves ecosystem services that benefit soil and animal health, as well as biodiversity. These systems enhance livestock diets by increasing the nutritional quality of forage and provide natural control over parasites (i.e., *H. irritans* and *Rhipicephalus microplus* ticks). As an environmental benefit, the greater abundance of dung beetles and earthworms facilitates the decomposition of dung pads, thereby reducing the *H. irritans* population [133]. Mariategui [134] found that increasing numbers of *Ontherus sulcator* (local dung beetle) relate to a decrease in the numbers of *H. irritans* larvae in feces, highlighting the importance of the use of the biocontrol of *H. irritans* in Argentina. Also, horn fly infestations and pupal density in dung pats were reduced in a silvopastoral system when compared to the single-pasture system. In comparison, the density of pupae was almost 4-fold higher in the former, suggesting that natural enemies may have contributed to reducing the horn fly population [135].

Plant products. Plant extracts and isolated compounds have been tested against several flies that cause primary (*C. hominivorax*) or secondary myiasis (*Lucilia* spp., *Musca* spp., etc.). A study conducted in Boyacá, Colombia, evaluated five plant extracts and found that *Nicotiana tabacum* achieved 100% mortality of *H. irritans* at its highest concentrations. Other extracts (i.e., *Brugmansia arborea* and *Sambucus nigra*) have also demonstrated insecticidal activity in vitro. This biofriendly approach could be a potential option for controlling insecticide-resistant *H. irritans* [136]. Although plant extracts can be used to control *H. irritans* in cattle, the availability of commercial products is still limited in LA.

Immunological control. The need for environmentally friendly and public health-friendly horn fly control methods has prompted research on the immune response of cattle to muscidae antigens. The concept is to develop anti-horn fly vaccines, a strategy analogous to the approach used for tick control. So far, a study has shown that 200 flies/animal elicit a weak antibody response to antigens in fly saliva. The data revealed that this effect increases when the flies are removed from the animals. This suggests the modulation of antigens in the *H. irritans* salivary glands [137].

Research is still needed to identify and test candidate antigens (i.e., thrombostasin, a coagulation-inhibiting protein) against *H. irritans* [138]. Although promising vaccine candidates against horn flies were identified by reverse vaccinology [139], the immunological control of *H. irritans* does not seem to be an alternative in the short term.

### 11.2. Non-Chemical Control of Dermatobia hominis

The oldest method of dermatobiosis control, manual larval extraction, may be an alternative for small farms and organic cattle farming systems, where the use of antiparasitic drugs is not permitted or restricted [140]. Although a commercial vaccine is not available on the Brazilian market, cattle immunized with an antigenic extract prepared from *D. hominis* larvae showed increased antibody production and a lower number of skin nodules (29 vs. 79 nodules) compared to cattle in the control group [141]. However, the immunological control of *D. hominis* is not a viable alternative in the short term.

### 11.3. Non-Chemical Control of C. hominivorax

Despite the success of the SIT (sterilization insect technique) in eradicating *C. hominivorax* in North America and Central America, there is no plan based on this tool as a complement to chemical control in the countries of the Southern Cone [142]. 

Uruguay recently implemented a plan to eradicate *C. hominivorax* throughout the national territory [143]. The SIT initiative is being intensified aiming the control of screwworm in areas of Central America and Mexico, where this species was eradicated few decades ago and COPEG (Panama-United States commission for the eradication and prevention of the cattle screwworm) has increased the production of sterile pupae from 20 to 90 million per week, fully directed to this New World screwworm outbreak. For this reason, Uruguay will have to find another source of sterile flies. Meanwhile, other control alternatives, such as the development of CRISPR/Cas9, which enables the editing of genomes to generate heritable modifications of specific desired characteristics, including reduced female fertility, are being explored [144].

## 12. Conclusions

- Cattle infesting flies cause large economic and health losses to animal production in LA, mainly due to stress, loss of energy, altered grazing behavior, decreased weight gain, low conversion efficiency, and/or skin damage, in addition to costs related to prevention and control.
- Within the antiparasitic market of LA, six classes of insecticides are available for fly control in cattle, SPs, MLs, OPs, PPs, IGRs, and isoxazolines, as well as mixtures of several insecticide classes. Brazil has the largest number of insecticide products (301) registered for fly control in cattle.
- *H. irritans* management has long relied on insecticides for prophylactic and therapeutic fly control, and a greater effort is needed in the search for alternatives.
- The economic problems caused by *H. irritans* infestations and the continuous increase in its resistance to insecticides threaten the success in the control currently being carried out, requiring an integrated management approach that aims at both fly control and resistance management.
- Although visually counting horn flies over the animals has been considered a theoretical criterion for determining how severe the infesting burden could be, the timing of cattle treatments, observing behavioral changes (such as frequency of head tosses) may be a more practical and useful approach for making decisions regarding tactical control.
- Efficacy failures and chemical insecticide resistance of *H. irritans*, *C. hominivorax* and *D. hominis* have been reported in LA.
- Although biological control using the natural enemies of these dipteran flies, such as parasitoids, entomopathogenic bacteria, nematodes, and fungi, has demonstrated some in vitro and field efficacy for the control of *H. irritans*, their commercial availability and use are very limited in LA.
- Advances in biological control, vaccination technologies, plant products, physical control, management systems, new insecticides (e.g., isoxazolines), repellents, and cattle genetics will significantly enhance producers’ ability to reduce losses due to ectoparasitic cattle flies.

## 13. Recommendations

- In temperate regions of LA where horn fly diapauses, such as Uruguay, the use of insecticides during the fall, to reduce horn fly populations whose progeny will become dormant, is recommended to decrease the abundance of spring adults.
- In controlling horn flies in LA, insecticide treatments and other measures should be strategically planned for periods of higher infestation levels but tactically implemented only if truly necessary.
- The timing of treatment is a decision for the producer. Although horn fly counts on animals are considered a (theoretical) criterion for insecticide treatments (e.g., >200 flies/animal), we recommend a more practical approach, carrying out tactical chemical treatments of the herd when approximately 25% of the animals exhibit head tossing behavior within one minute of observation. Special attention should be paid to bulls due to their high susceptibility to horn flies, and their selective insecticide treatment is recommended if necessary.
- For *D. hominis* control in tropical regions of LA, it is recommended to prevent infestation during the rainy season by administering MLs every two months. In clinical cases, it is advisable to apply topical OP products directly to the nodules, perform manual larval extraction, and administer ML 1% subcutaneously (0.2 mg/kg body weight).
- When *C. hominivorax* myiasis is detected on animals (mainly in cattle), it is recommended to implement local treatment with larvicidal products (e.g., chlorpyriphos, coumaphos, propoxur, dichlorvos, fenitrothion, alone or in mixture with SPs). Additionally, the administration of MLs (e.g., DRM), fipronil or isoxazolines is recommended.
- Immediate prophylaxis against *C. hominivorax* infestations following surgical interventions (e.g., castration, dehorning) or parturition is critical to prevent myiasis and associated morbidity. Systemic endectocides such as macrocyclic lactones (e.g., IVM, DRM), phenylpyrazoles (e.g., fipronil), and isoxazolines have demonstrated efficacy in preventing larval establishment when administered according to approved protocols.
- Although resistance is a concern in some locations and countries, to protect calves from umbilical myiasis caused by *C. hominivorax*, the preventive application of MLs by injection is recommended in the first few hours/days after birth. To delay the development of resistance in *H. irritans* and *C. hominivorax*, it is recommended to rotate insecticides with different modes of action—which means alternating insecticide classes, considering the classes currently available on the LA market—and avoid using products from insecticide classes that have not demonstrated the expected efficacy.
- Strict adherence to the manufacturer’s label instructions for veterinary medicinal products is essential to ensure therapeutic efficacy and minimize risks to animal health, human safety, and the environment. Deviations from approved use, such as incorrect dosing, off-label administration, or failure to observe withdrawal periods, can result in adverse effects, including toxicity, antiparasitic resistance, and chemical residues in food products

## Figures and Tables

**Table 1:** Chemical groups available in Latin America with activity on *Dermatobia hominis* (larvicidal activity on animal), *Cochliomyia hominivorax* (larvicidal activity on animal), and *Haematobia irritans* (adult activity on animal and/or larval activity in feces).

Chemical Group	Administration Forms	Fly Control	Countries
**Insect growth regulator**			
Diflubenzuron 3%	Oral added to food	*H. irritants* *	Argentina, Brazil, Mexico
Diflubenzuron 25%	Oral added to food	*H. irritants* *	Brazil
**Isoxazolines**			
Fluralaner 5%	Pour-on	*D. hominis*, *C. hominivorax*, *H. irritans*	Argentina, Brazil, Mexico, Uruguay
Fluralaner 2.5%	Pour-on	*D. hominis*, *C. hominivorax*, *H. irritans*	Brazil
**Macrocyclic lactones**			
Ivermectin 0.8%	Injectable	*D. hominis*	Brazil
Ivermectin 0.5%	Injectable	*D. hominis*	Brazil
Ivermectin 1%	Injectable, pour-on	*D. hominis*, *C. hominivorax*, *H. irritans*	Argentina, Brazil, Colombia, Mexico, Uruguay
Ivermectin 3.15%	Injectable	*D. hominis*, *C. hominivorax*, *H. irritans*	Mexico, Uruguay, Brazil
Ivermectin 3.25%	Injectable	*D. hominis*, *C. hominivorax*	Brazil
Ivermectin 3.5%	Injectable	*D. hominis*, *C. hominivorax*	Brazil
Ivermectin 3.6%	Injectable	*D. hominis*	Brazil
Ivermectin 3.75%	Injectable	*D. hominis*	Brazil
Ivermectin 4%	Injectable	*D. hominis*	Brazil
Abamectin 0.5%	Injectable, pour-on	*D. hominis*, *H. irritans*,	Brazil
Abamectin 1.13%	Injectable	*D. hominis*, *C. hominivorax*, *H. irritans*	Argentina
Abamectin 1%	Injectable, pour-on	*D. hominis*, *C. hominivorax*, *H. irritans*	Brazil
Abamectin 2%	Injectable	*C. hominivorax*	Brazil
Doramectin 0.5%	Injectable	*D. hominis*, *C. hominivorax*	Brazil
Doramectin 1%	Injectable	*D. hominis*, *C. hominivorax*, *H. irritans*	Argentina, Brazil, Colombia, Mexico, Uruguay
Doramectin 1.1%	Injectable	*D. hominis*, *C. hominivorax*	Brazil
Doramectin 3.13%	Injectable	*D. hominis*, *C. hominivorax*, *H. irritans*	Argentina, Uruguay
Doramectin 3.15%	Injectable	*D. hominis*, *C. hominivorax*	Brazil
Doramectin 3.5%	Injectable	*D. hominis*, *C. hominivorax*, *H. irritans*	Brazil
Moxidectin 1%	Injectable	*D. Hominis*, *H. irritans*	Colombia, Mexico
Abamectin 8% + piperonyl butoxide	Impregnated ear tag	*H. irritans*	Uruguay
Eprinomectin 0.5%	Pour-on, injectable	*H. irritans*, *D. hominis*	Argentina, Brazil
Eprinomectin 0.6%	Pour-on	*H. irritans*, *D. hominis*	Brazil
Eprinomectin 1%	Injectable, pour-on	*H. irritans*, *D. hominis*	Brazil
Eprinomectin 2%	Injectable	*H. irritans*, *D. hominis*	Brazil
Eprinomectin 4.8%	Injectable	*C. hominivorax*, *H. irritans*	Brazil
Eprinomectin 5%	Injectable	*D. hominis*, *C. hominivorax*, *H. irritans*	Brazil
**Organophosphates**			
Coumaphos 20%	Spray	*H. irritans*	Mexico
Chlorpyriphos 5%	Spray	*C. hominivorax*	Brazil
Diazinon 21.4%	Impregnated ear tag	*H. irritans*	Colombia, Argentina, Mexico
Diazinon 40%	Impregnated ear tag	*H. irritans*	Argentina, Brazil, Uruguay
Diazinon 45%	Impregnated ear tag	*H. irritans*	Brazil
Diazinon 60%	Impregnated ear tag	*H. irritans*	Brazil
Diazinon 30% + chlorpyriphos 10%	Impregnated ear tag	*H. irritans*	Argentina, Brazil, Mexico
Diazinon 36% + fipronil 4%	Impregnated ear tag	*H. irritans*	Brazil
Diazinon 4%	Pour-on	*C. hominivorax*	Uruguay
Ethion 15%	Impregnated ear tag	*H. irritans*	Colombia, Mexico
Ethion 40%	Impregnated ear tag	*H. irritans*	Argentina, Brazil
Fenitrothion 6.63%	Pour-on, spray	*C. hominivorax*	Brazil, Uruguay
Fenitrothion 6.97%	Pour-on	*C. hominivorax*	Argentina, Uruguay
Fenitrothion 5%	Paste	*C. hominivorax*	Argentina
Fenthion 15%	Pour-on	*D. hominis*, *C. hominivorax*	Argentina
Trichlorfon 0.75%	Unguent	*D. hominis*, *C. hominivorax*	Brazil
Trichlorfon 30%	Pour-on	*D. hominis*, *C. hominivorax*, *H. irritans*	Brazil
Trichlorfon 50%	Pour-on, spray	*D. hominis*, *C. hominivorax*, *H. irritans*	Brazil
Trichlorfon 98%	Spray, oral	*D. hominis*, *C. hominivorax*, *H. irritans*	Brazil
Fenthion 15%	Spot-on	*D. hominis*, *C. hominivorax*, *H. irritans*	Brazil
Dichlorvos 74.45%	Spray	*D. hominis*	Brazil
**Phenylpyrazoles**			
Fipronil 0.32%	Spray	*D. hominis*, *C. hominivorax*	Brazil
Fipronil 0.7%	Pour-on	*C. hominivorax*	Argentina, Uruguay
Fipronil 1%	Pour-on	*D. hominis*, *C. hominivorax*, *H. irritans*	Argentina, Brazil, Colombia, Mexico
Fipronil 3%	Pour-on	*D. hominis*, *C. hominivorax*, *H. irritans*	Brazil
Fipronil 3.2%	Pour-on	*C. hominivorax*	Argentina, Uruguay
**Synthetic pyrethroids**			
Alfa-cypermethrin 1.5% + piperonyl butoxide 6%	Pour-on	*H. irritans*	Argentina, Uruguay
Alfa-cypermethrin 3%	Pour-on	*H. irritans*	Argentina, Uruguay
Alfamethrin 1%	Pour-on	*H. irritans*	Argentina, Uruguay
Cypermethrin 0.3%	Pour-on, topical solution	*C. hominivorax*	Argentina, Uruguay
Cypermethrin 2.5%	Pour-on	*H. irritans*	Argentina, Uruguay
Cypermethrin 3%	Paste	*C. hominivorax*	Argentina
Cypermethrin 15%	Pour-on, spray, dip	*D. hominis*, *H. irritans*	Argentina, Brazil, Colombia, Mexico, Uruguay
Cypermethrin 5%	Pour-on	*H. irritans*	Brazil, Uruguay, Argentina,
Cypermethrin 6%	Pour-on, spray	*H. irritans*	Argentina, Brazil, Uruguay
Cypermethrin 6% + piperonyl butoxide 7%	Pour-on	*H. irritans*	Argentina, Uruguay
Cyhalothrin 1%	Unguent	*D. hominis*, *C. hominivorax*	Brazil
Deltamethrin 3%	Dip	*H. irritans*	Argentina, Uruguay
Deltamethrin 25%	Spray, dip	*D. hominis*, *H. irritans*	Brazil, Colombia, Mexico
Flumethrin 3%	Pour-on, spray, dip	*D. hominis*, *H. irritans*	Mexico
Lambda-cyhalothrin 3%	Pour-on	*H. irritans*	Argentina, Uruguay
Permethrin 0.5%	Unguent, spray	*D. hominis*, *C. hominivorax*	Brazil
**Mixtures of insecticides**			
Coumaphos 2% + propoxur 1.5%	Powder	*C. hominivorax*	Argentina, Mexico
Cypermethrin 15% + chlorpyriphos 25%	Spray, dip	*H. irritans*	Colombia, Brazil, Mexico
Cypermethrin 15% + chlorpyriphos 25% + piperonyl butoxide 1%	Spray	*H. irritans*, *D. hominis*	Brazil
Cypermethrin 15% + chlorpyriphos 25% + piperonyl butoxide 15%	Spray	*H. irritans*, *D. hominis*	Brazil
Cypermethrin 15% + chlorpyriphos 30% + fenthion 15%	Spray	*H. irritans*, *D. hominis*	Brazil
Chlorpyriphos 24% + permethrin 5%	Spray, dip	*H. irritans*	Colombia, Mexico
Cypermethrin 5% + ethion 15%	Pour-on	*H. irritans*, *D. hominis*	Argentina, Brazil
Cypermethrin 5% + trichlorphon 1%	Paste	*C. hominivorax*	Argentina
Cypermethrin 15% + chlorpyriphos 25%	Pour-on	*D. hominis*	Colombia, Mexico
Cypermethrin 2% + chlorpyriphos 0.4%	Pour-on	*C. hominivorax*	Argentina, Uruguay
Cypermethrin 2% + chlorpyriphos 2%	Pour-on	*C. hominivorax*	Argentina, Uruguay
Cypermethrin 2% + dichlorvos 1%	Pour-on, paste	*C. hominivorax*	Argentina
Cypermethrin 3% + imidacloprid 0.7%	Pour-on, paste	*C. hominivorax*	Argentina, Uruguay
Cypermethrin 0.15% + chlorfenviphos 2.5%	Powder	*D. hominis*, *C. hominivorax*	Brazil
Cypermethrin 0.4% + dichlorvos 1.6% + silver sulfadiazine + piperonyl butoxide	Spray	*C. hominivorax*	Argentina
Cypermethrin 0.4% + chlorfenviphos 1.6%	Spray	*D. hominis*, *C. hominivorax*	Brazil
Cypermethrin 0.4% + dichlorvos 1.6%	Spray	*D. hominis*, *C. hominivorax*	Brazil
Cypermethrin 0.4% + fenitrothion 0.5%	Pour-on	*C. hominivorax*	Argentina
Cypermethrin 0.5% + dichlorvos 1% + trichlorfon 2%	Topical solution	*D. hominis*, *C. hominivorax*	Brazil
Cypermethrin 0.5% + dichlorvos 1.8%	Spray	*C. hominivorax*	Brazil
Cypermethrin 4% + imidacloprid 4% + PPB 4%	Pour-on	*C. hominivorax*	Argentina
Cypermethrin 0.37% + dichlorvos (dichlorovinyl dimethyl phosphate) 1.6%	Spray	*C. hominivorax*	Argentina
Cypermethrin 0.1% + fenitrothion 0.5%	Spray	*C. hominivorax*	Argentina
Cypermethrin 1% + carbaryl 2%	Powder	*C. hominivorax*	Argentina, Brazil
Cypermethrin 1.625% + imidacloprid 0.82%	Spray	*C. hominivorax*	Brazil
Imidacloprid 0.15 + etofenprox 0.15%	Spray	*C. hominivorax*	Argentina
Cypermethrin 0.5% + diazinon 1.92%	Pour-on	*C. hominivorax*	Uruguay
Imidacloprid 0.70% + cypermethrin 4%	Pour-on	*C. hominivorax*	Uruguay
Abamectin 1% + cypermethrin 6%	Pour-on	*H. irritans*	Uruguay
Cypermethrin 5% + chlorpyriphos 2.5%	Pour-on	*D. hominis*, *H. irritans*	Argentina, Brazil, Uruguay
Cypermethrin 2% + imidacloprid 2%	Topical solution	*C. hominivorax*	Brazil
Cypermethrin 4% + imidacloprid 4% + fluazuron 3%	Pour-on	*D. hominis*, *C. hominivorax*, *H. irritans*	Brazil
Cypermethrin high cis 5% + chlorpyriphos 2.5% + piperonyl butoxide 1%	Pour-on	*D. hominis*, *H. irritans*	Brazil
Cypermethrin 5% + chlorpyriphos 7%	Pour-on	*D. hominis*, *H. irritans*	Brazil
Cypermethrin 5% + chlorpyriphos 7% + piperonyl butoxide 1%	Pour-on	*D. hominis*, *H. irritans*	Brazil
Cypermethrin 5% + chlorpyriphos 7% + piperonyl butoxide 5%	Pour-on	*D. hominis*, *H. irritans*	Brazil
Cypermethrin 5% + chlorpyriphos 7% + fluazuron 2.5% + piperonyl butoxide 5%	Pour-on	*D. hominis*, *H. irritans*	Brazil
Cypermethrin 5% + dichlorvos 45%	Spray	*D. hominis*, *H. irritans*	Brazil
Cypermethrin 5% + trichlorfon 30% + piperonyl butoxide 15%	Spray	*D. hominis*, *H. irritans*	Brazil
Cypermethrin 5% + fenitrothion 4% + piperonyl butoxide1%	Pour-on	*H. irritans*	Argentina
Cypermethrin 5% + trichlorfon 10% + piperonyl butoxide 5%	Pour-on	*H. irritans*	Argentina, Uruguay
Cypermethrin 5% + carbaryl 2% + piperonyl butoxide 7%	Pour-on	*H. irritans*	Uruguay
Cypermethrin 5.5% + fenitrothion 0.75%	Pour-on	*H. irritans*	Brazil
Cypermethrin 6% + chlorpyriphos 7% + piperonyl butoxide 0.5%	Pour-on	*D. hominis*, *H. irritans*	Brazil
Cypermethrin 6% + piperonyl butoxide 7% + carbaryl 2%	Pour-on	*H. irritans*	Argentina, Uruguay
Cypermethrin 6% + chlorpyriphos 7%	Pour-on	*H. irritans*	Brazil
Cypermethrin high cis 6% + chlorpyriphos 50%	Spray	*D. hominis*, *H. irritans*	Brazil
Cypermethrin 6% + chlorpyriphos 7% + piperonyl butoxide 5% + fluazuron 3%	Pour-on	*D. hominis*, *H. irritans*	Brazil
Cypermethrin 7.5% + chlorpyriphos 12.5% + piperonyl butoxide 30%	Spray	*D. hominis*, *H. irritans*	Brazil
Cypermethrin + 20% + chlorpyriphos 50%	Spray	*H. irritans*, *C. hominivorax*	Argentina, Brazil, Uruguay
Prallethrin 0.5% + trichlorfon 2%	Spray	*C. hominivorax*	Brazil
Fipronil 0.9% + abamectin 0.5%	Pour-on	*D. hominis*, *C. hominivorax*, *H. irritans*	Argentina, Brazil, Uruguay
Dichlorvos 60% + chlorfenvinphos 15%	Spray	*D. hominis*	Brazil
Dichlorvos 60% + chlorfenvinphos 20%	Spray	*D. hominis*, *C. hominivorax*, *H. irritans*	Brazil
Dichlorvos 1% + trichlorfon 2%	Topical solution	*C. hominivorax*	Brazil
Fenthion 15% + fluazuron 2.5%	Pour-on	*H. irritans*, *D. hominis*	Brazil
Chlorpyriphos 7% + fluazuron 2.5%	Pour-on	*D. hominis*, *C. hominivorax*, *H. irritans*	Brazil
Chlorpyriphos 0.71%+ dichlorvos 1.15%	Pour-on	*C. hominivorax*	Uruguay
Diazinon 2.15% + cyromazine 0.05%	Pour-on	*C. hominivorax*	Uruguay
Diazinon 2% + trichlorfon 1.5% + sulfathiazole 1%	Pour-on	*C. hominivorax*	Uruguay
Dichlorvos 0.83% + chlorfenvinphos 0.52%	Pour-on, spray	*C. hominivorax*	Argentina, Uruguay
Eprinomectin 4.8% + fluazuron 10%	Injectable	*H. irritans*	Brazil
Ivermectin 3.15% + fluazuron 8%	Injectable	*D. hominis*, *C. hominivorax*	Brazil
Eprinomectin 1.8% + fluazuron 8%	Injectable	*H. irritans*	Brazil
Doramectin 1% + eprinomectin 1%	Injectable	*D. hominis*	Brazil
Moxidectin 1% + eprinomectin 1%	Injectable	*D. hominis*	Brazil
Ivermectin 2% + abamectin 1.2% + doramectin 1%	Injectable	*D. hominis*, *C. hominivorax*	Brazil
Ivermectin 2.25% + abamectin 1.25%	Injectable	*D. hominis*	Brazil
Abamectin 0.5% + fluazuron 2.5%	Pour-on	*C. hominivorax*, *H. irritans*, *D. hominis*	Brazil
Abamectin 0.6% + fluazuron 3%	Pour-on	*H. irritans*	Brazil
Abamectin 5% + fluazuron 3%	Pour-on	*D. hominis*, *H. irritans*	Brazil
Fipronil 1% + fluazuron 2.5%	Pour-on	*D. hominis*, *C. hominivorax*, *H. irritans*	Brazil
Fipronil 1% + fluazuron 3%	Pour-on	*D. hominis*, *C. hominivorax*, *H. irritans*	Brazil
Fipronil 1.25% + fluazuron 2.5%	Pour-on	*D. hominis*, *C. hominivorax*, *H. irritans*	Brazil
Fipronil 1.25% + eprinomectin 0.5% + fluazuron 3%	Pour-on	*D. hominis*, *C. hominivorax*, *H. irritans*	Brazil
Fipronil 1.5% + fluazuron 8%	Spray	*D. hominis*, *C. hominivorax*, *H. irritans*	Brazil
Fipronil 5% + fluazuron 12.5%	Pour-on	*H. irritans*	Brazil
Diazinon 30% + chlorpyriphos 10%	Impregnated ear tag	*H. irritans*	Argentina, Mexico, Uruguay
Cypermethrin 5% + ethion 15%	Impregnated ear tag	*H. irritans*	Colombia, Mexico

*: larval activity in feces.

## Data Availability

No new data were created or analyzed in this study.

## Abbreviations

The following abbreviations are used in this manuscript:

OPs	Organophosphates
SPs	Synthetic pyrethroids
PPs	Phenylpyrazolones
IGRs	Insect growth regulators
MLs	Macrocyclic lactones
*H. irritans*	*Haematobia irritans*
*D. hominis*	*Dermatobia hominis*
*C. hominivorax*	*Cochliomyia hominivorax*
DRM	Doramectin
IVM	Ivermectin
MOX	Moxidectin
DPT	Days post-treatment

## Appendix A. Life Cycle and Main Biological Characteristics of Cattle Infesting Flies

### Appendix A.1. Haematobia irritans (Horn Fly)

The horn fly is a dipteran belonging to the family Muscidae. The common names in Latin America are “mosca de los cuernos” and “mosca-dos-chifres”. This fly is a widely distributed ectoparasite of cattle that negatively affects cattle production. The direct effects of *H. irritans* on cattle include constant restlessness, skin damage, and blood loss, which cause a reduction in the production of milk and meat. The impact of *H. irritans* on animal production is related to parasite burden, which depends on animal characteristics and regional environmental conditions [8]. Adults spend most of their lives on the host and tend to congregate on the back and shoulders of cattle or underbelly at the hottest times of the day [15].

The ovigerous female is found in the ventral area and on the legs of the bovine, close to depositing the eggs in the fecal matter, with this being the only substrate that allows for larval development. Desiccations, trampling of animals, and disintegration due to rain affect immature viability. Females leave the host to lay eggs in fresh dung, and a single female can lay up to 360–400 eggs in batches of up to 14–20 eggs; egg hatching occurs in less than 24 h [16]. Larvae go through three developmental stages in 3 to 5 days, and the pupal stage lasts for about the same period until adult emergence [15].

In temperate regions, pupae can stop their cycle and slow their metabolism, a phenomenon called diapause. This characteristic helps maintain fly populations in refuge, which would begin 30 days before the end of the fly season, and is induced by temperatures below 12–15 °C. To exit diapause, the temperature must be above 18 °C for a minimum of 14 days [17].

Another characteristic of this species of fly is that the males can copulate with five to eight females, while the female mates only once in her lifetime, which is generally carried out over the bovine body surface. Flies copulate on the second or third day after birth and no later than the fifth day [15]. The complete life cycle from egg to adult (Figure 1) may range from just 7 to 11 days to more than 4 weeks [18] depending on temperature. In Uruguay, 12 to 15 generations of flies are produced per year, while in Brazil, there can be 19 (southeast) to 30 (northeast) generations yearly [19,20]. After emergence, adults search immediately for a host to feed.

Most flies are located on the dorsal region of the bovine, adopting a typical position with the head directed ventrally and its wings semi-open, forming an angle of 60 -degree (delta wing shape). On very hot or windy days, it is mostly located in more sheltered areas, e.g., in the abdomen. Flies move away from the bovine when entering a dark place (e.g., when entering a milking parlor). Adult flies remain on cattle most of the time, feeding on their blood and tissue fluids several times a day (26–35 times). The fly does not survive more than 26 h without feeding [8].

There is a preference of the fly for black fur over white. Likewise, animals with thick, long fur are less attacked than those with short fur. Zebu breeds are less likely to be parasitized due to the greater mobility of the cutaneous muscle and the fact that the fecal matter has less texture and forms a thin layer that is easy to desiccate compared to European breeds [20].

In a study on parasite–host interaction, it has been determined that the sucking apparatus of hematophagous insects, when interacting with the skin of the host, produces tissue damage that causes an inflammatory reaction, with the “complement system” being able to participate through the sequential activation of its proteins, whose main effects are the lysis of target cells and tissue inflammation [8]. The fly counteracts this reaction with anticoagulant and anti-inflammatory substances. Some of these substances, such as thrombostasin (mammalian thrombin inhibitor), have been isolated from the salivary gland of *H. irritans* [21].

Harris et al. [22] noted that under experimental conditions, female horn flies spent an average of 163 min/day feeding; males averaged 96 min/day. Each female ingested an average of 17.1 mg of blood per day, whereas males ingested 12.1 mg per individual because of the difference in feeding times [23].

The irritation caused by feeding reduces grass consumption; interferes with the rumination, digestion, and assimilation of food; and increases energy expenditure due to the intense and frequent defensive behaviors of infested animals, like the head tossing [8].

Horn flies have a varying preference for cattle of different coat colors, breeds, genders, and ages, being greater for adult dark-haired males [20]. Horn flies are more attracted to bulls than cows or steers [24]. It is speculated that the increased attractiveness of these animals might be due to the physiological effect of testosterone on the sebaceous glands and their secretions [16]. Horn fly infestations increase with the age of the animals and cause economic losses, significantly reducing the weight gain of Nelore cattle from weaning to the age of slaughter [9]. In general, calves have less flies than adults.

### Appendix A.2. Dermatobia hominis (Bot Fly)

The bot fly is a dipteran belonging to the family Oestridae, subfamily Cuterebrinae. Its common names in Latin American countries are “Tórsalo”, “Rezno”, “Ura”, “Berne”, “Nuche”, “Colmoyote”, and “Moyocuil”. The adult fly has atrophied mouthparts, and therefore it does not feed [25]. Females lay eggs on phoretic flies [12] by using their legs to immobilize the wings of the phoretic arthropod and attaches between 15 and 30 eggs to its abdomen. The process is repeated successively since it is capable of producing between 100 and 400 eggs during its 8–9 days of life. The phoretic arthropod is responsible for carrying the eggs to their definitive host [26]. These intermediate arthropods vary according to the microclimate and geographic location of each region. The main ones are mosquitoes of the genus *Psorophora* or the *Stomoxys* fly, followed by mosquitoes of the genus *Aedes*, other flies (*H. irritans*, *Musca domestica*), and horse flies [27]. For cattle, the most important species are *H. irritans*, *M. domestica*, and *S. calcitrans* in the transport of the eggs of the *D. hominis* fly [13].

When the phoretic arthropod lands on the host’s skin, its body heat stimulates the hatching of the larva, which penetrates the skin using oral hooks at the anterior end. It penetrates through small wounds (warble), at the bite site of the phoretic arthropod or through intact skin via the hair follicle. This process does not cause pain and takes between 5 and 60 min [28].

L1 remains close to the entry site, where it feeds on the surrounding soft tissues. It then penetrates the skin into a subdermal cavity and keeps the initial hole open to breathe. Inside the cavity, the larva feeds and molts twice to transform into L2 and L3 after 5–10 weeks. During the development period, a series of concentric and parallel lateral spines with an antero-posterior orientation appears, along with their oral hooks. During this period, painful nodules develop for the animal or human host. After complete development, the L3 larva leaves the host, leaving a hole in the skin, resulting in hide damage (Figure 2). L3 leaves the host at night or at the beginning of the day, never in the afternoon, probably to avoid desiccation [13].

L3 falls to the ground, buries itself, and pupates. Adults emerge from the puparium 3–5 weeks later, depending on weather conditions. After this period, an imago or young fly is produced, which, in a few hours, is then fertilized to start the life cycle again. Adult flies do not feed and use energy reserves accumulated in the larval stage [13].

### Appendix A.3. Cochliomyia hominivorax (New World Screwworm)

*C. hominivorax* is a dipteran belonging to the family Calliphoridae and subfamily Chrysomyinae. Its common names in Latin America are “gusano barrenador del ganado”, “mosca de la carne”, and “mosca-da-bicheira”. It is a parasite of mammals, including humans, during its larval stages. The larvae penetrate the host’s body through natural or husbandry-related wounds, as well as via the mucous membranes of bodily orifices [14]. Larvae feeding on the skin and underlying tissues of the host cause a condition known as wound or traumatic myiasis, which can be fatal.

Blood odor and secretions from the inflammatory process of animals and humans attract screwworm flies. The females lay 250–500 eggs in the exposed flesh of warm-blooded animals and humans, such as in wounds and the navels of newborn animals. Within 24 h, larvae emerge and burrow deeply into the wound. Larvae (L1) hatch from the eggs and feed on the wound, continuing their development into L2 and L3. The maggots can cause severe tissue damage or even death to the host. After 5 to 7 days, the L3 larva leaves the wound and falls in the soil to pupate. Environmental and wound temperatures influence the development rate of flies in immature stages, being slower at low temperatures. The pupal stage can last from one week to 2 months in the soil until adult emergence. Female flies mate once in their lifetime, about four to five days after emerging. The entire life cycle lasts about 20 days. Adults can fly up to 200 km (120 mi) during their life [14] (Figure 3).

## Appendix B. Current Situation of *Cochliomyia hominivorax* Distribution in Latin America

Production and release of sterile flies. The Sterile Insect Technique (SIT) is a unique methodology that is applied in an area to eliminate the existing or residual population of *C. hominivorax* that remains after implemented control activities such as epidemiological surveillance, wound treatment of infested lesions, the control of animal transport, or quarantine [14]. When a male’s sperm is irradiated (e.g., via X-rays), cytogenetic consequences occur where the chromosomes break, and during cell division, the daughter cells may not receive all of the genetic information needed to function. These chromosomal events culminate in the death of any embryo formed by the fertilization of a normal egg cell with irradiated sperm (https://www.fao.org/4/u4220t/u4220T0j.htm, accessed on 1 January 2026).

New World screwworm sterile flies are produced in plants or factories where the temperature and humidity conditions in which the adult fly lives in nature are artificially reproduced, as well as when it parasitizes wounds. In artificial breeding, many animals originated raw materials such as blood meal, milk, and others are used. The operation occurs continuously 24 h a day throughout the year. When the pupae are 5½ days old, they are cast into irradiators where they are exposed to gamma radiation with Cesium 136 or to X-rays at the minimum dose to achieve sexual sterility, both in males and females, without producing any adverse effect on their biological quality to compete with wild ones. When mass-produced, the release of these sterile flies results in sterile male flies mating with wild female flies which then lay unfertilized eggs. Since female *C. hominivorax* normally mate only once, its population of is progressively reduced and, ultimately, eradicated [14].

Nowadays there is only one plant producing sterile flies worldwide, and it belongs to the Panama-United States Commission for the Eradication and Prevention of Screwworms (COPEG), which currently produces 20 million sterile flies per week (it can produce up to 100 million/week). In 2020, COPEG reported that 16 million sterile insects were released in the border area between Colombia and Panama, and 4 million were dispersed in the vicinity of the plant to mitigate the risk of the escape of fertile flies. They are subsequently sent to dispersal centers from where they are released a day or two after sterilization. The release methods are as follows: (a) the release of flies in cardboard boxes in small planes; (b) the release of insects in bulk through the Cold Dormant Fly System, with the use of a dispensing machine from small planes; (c) terrestrial sterile fly release stations; and (d) the terrestrial dispersal of sterile flies through automotive vehicles [14].

NWS eradication. In order to eradicate the NWS, it is necessary to at least establish the following measures: (a) the detection and reporting of larval samples suspected of being *C. hominivorax* larvae by farmers and pet owners, with the purpose of obtaining daily information on the incidence and distribution of the parasite; (b) the control of mobilization and quarantine of animals, in order to avoid the spread of the disease; (c) preventive and curative treatments of wounds managed constantly and daily by farmers; (d) the use of sterile adult *C. homivorax* flies; and (e) the implementation of an extensive educational campaign on the progress of the program and how animal owners can reinforce NWS control [100].

Countries where NWS has been eradicated. The political and technical will of several countries, diplomatic efforts and the implementation of successful international technical cooperation programs, occasioned success on the eradication of *C. hominivorax* in the following countries in the American Continent [14]:

- North America: United States of America (1966) and Mexico (1991).
- Central America: Belize (1994), El Salvador (1995), Guatemala (1994), Honduras (1996), Nicaragua (1998), Costa Rica (2000), and Panama (2006).
- The Caribbean: Aruba (1954), Bonaire (1954), Curacao (1954, 1976 reinfestation), Puerto Rico (1975), US Virgin Islands (1971), and British Virgin Islands (1972).
- América del sur: Chile (1947 and in 2017 after a reintroduction episode).

Countries where NWS presence to our knowledge has never been reported are:

- North America: Canada.
- The Caribbean: Antigua and Barbuda, Barbados, Bahamas, Dominica, Cayman Islands, Grenada, Guadeloupe, Saint Kitts and Nevis, Martinique, Montserrat, Saint Vincent and the Grenadines, Saint Lucia, and Turks and Caicos.

Countries with NWS presence. In 2020, the geographical distribution of GBG was located endemically in the following countries:

- The Caribbean: Cuba, Haiti, Jamaica, Dominican Republic, and Trinidad and Tobago.
- South America: Argentina, Bolivia, Brazil, Colombia, Ecuador, Guyana, French Guiana, Paraguay, Peru, Suriname, Uruguay, and Venezuela.

Resurgence of NWS in the Americas. Central America had been free of NWS since 2006; however, in February 2023, Panama declared an outbreak of this fly in the Darién province. Subsequently, in early July 2023, the Ministry of Agricultural Development of Panama declared a state of emergency due to the increase in *C. hominivorax* infestation cases. Subsequently, on 14 July 2023, the National Animal Health Service of Costa Rica reported the first detection of NWS in its territory. On 13 September 2024, authorities from the Ministry of Agriculture and Livestock of Honduras confirmed six cases of NWS in horses and cattle that had entered the country irregularly. Subsequently, on 29 October 2024, the Ministry of Agriculture, Livestock and Food of Guatemala reported the first case of NWS in the country, and a state of animal health emergency was declared [100]. Mexico reported the resurgence of NWS in November 2024.

Potential causes and/or risk factors for the resurgence of NWS in eradicated Central American countries and México. The resurgence and spread of NWS to Central American countries and Mexico can have occurred due to single and/or multiple combined factors as mentioned below [103,104].

- Insufficient dispersal of sterile flies in Panama by COPEG, where only 20 mill flies/week were routinely produced, of which 16 mill were released in the border area between Colombia and Panama and 4 mill/week in the vicinity of the sterile fly production plant to mitigate the risk of the escape of fertile flies from the colony.
- Ineffective monitoring of the distribution of released sterile flies in the Darien province (i.e., by the use of sticky traps baited with liver bait stations).
- Loss of *C. hominivorax* strain effectiveness in the field as a result of long-term colonization.
- The fly’s ability to travel great distances. Its dispersion can be favored by strong air currents. It has been recorded that a fly can fly up to 290 km.
- Transport of live animals with screwworms or through skin hides without inspection.
- Transport of hunting trophies from an endemic area to a free area.
- Transport of vehicles that use beds to transport animals and that may harbor viable larvae or pupae.
- Favorable conditions for the development of screwworm fly in Central American countries (high temperatures and humidity).
- Lack of infrastructure (mainly for small producers) for the management of animals and to identify infestations and effectively treat screwworm larvae.
- Recurrent fights between animals (domestic and wild).
- Cross-border dislogement of wild animals with screwworms.
- Cross-border transport of infested pets by their owners without veterinary inspection.
- Unregulated and/or illegal movement of infested livestock, which are not inspected by trained veterinarians.
